# A genomic-led strategy to anticipate drug safety effects

**DOI:** 10.1371/journal.pgen.1012211

**Published:** 2026-07-16

**Authors:** Brian R. Ferolito, Andrea R. V. R. Horimoto, Kai Gravel-Pucillo, Daniel J. Golden, Hesam Dashti, Claudia Giambartolomei, Danielle Rasooly, Rachael Matty, Liam Gaziano, Yakov Tsepilov, Lauren Costa, Nicole Kosik, Harris Ioannidis, Mohd Karim, Giovanna Winicki, Fiona Hunter, Claudia Langenberg, John C. Whittaker, Million Veteran Program, Tianxi Cai, Gina M. Peloso, Barbara Zdrazil, Maya Ghoussaini, Andrew R. Leach, Sumitra Muralidhar, Ines A. Smit, Juan P. Casas, J. Michael Gaziano, Kelly Cho, Alexandre C. Pereira

**Affiliations:** 1 Million Veteran Program (MVP) Coordinating Center, Veterans Affairs Healthcare System, Boston, Massachusetts, United States of America; 2 Department of Genetics, University of North Carolina School of Medicine, Chapel Hill, North Carolina, United States of America; 3 Division of Aging, Brigham and Women’s Hospital and Harvard Medical School, Boston, Massachusetts, United States of America; 4 The Novo Nordisk Foundation Center for Genomic Mechanisms of Disease, Broad Institute of MIT and Harvard, Cambridge, Massachusetts, United States of America; 5 Health Data Science Centre, Human Technopole, Milan, Italy; 6 Centre for Human Technologies (CHT), Istituto Italiano di Tecnologia, Genova, Italy; 7 BHF Cardiovascular Epidemiology Unit, University of Cambridge, Cambridge, United Kingdom; 8 Open Targets Genetics, Open Targets, Hinxton, United Kingdom; 9 European Molecular Biology Laboratory, European Bioinformatics Institute, Hinxton, United Kingdom; 10 Genomic Discovery, Variant Bio, Seattle, Washington, United States of America; 11 Precision Healthcare University Research Institute, Queen Mary University of London, London, United Kingdom; 12 Computational Medicine, Berlin Institue of Health at Charit´e – Universitätsmedizin, Berlin, Germany; 13 MRC Epidemiology Unit, University of Cambridge, Cambridge, United Kingdom; 14 MRC Biostatistics Unit, University of Cambridge, Cambridge, United Kingdom; 15 Department of Biomedical Informatics, Harvard Medical School, Boston, Massachusetts, United States of America; 16 Department of Biostatistics, Boston University School of Public Health, Boston, Massachusetts, United States of America; 17 Regeneron Genetics Center, Regeneron Pharmaceuticals, Tarrytown, New York, United States of America; 18 LifeArc, Accelerator Building, Open Innovation Campus, Stevenage, United Kingdom; 19 Department of Veterans Affairs, Office of Research and Development, Washington District of Columbia, United States of America; 20 Biomarker Development/Translational Medicine, Novartis Institutes for Biomedical Research, Cambridge, Massachusetts, United States of America; 21 Department of Medicine, Harvard Medical School, Boston, Massachusetts, United States of America; Stanford University, UNITED STATES OF AMERICA

## Abstract

Safety-related issues account for approximately 25% of failures in new drug discovery programs. On top of that, many are discovered during post-marketing surveillance, significantly limiting drug utility and application. To proactively address these concerns, we developed a genetics-led strategy leveraging Mendelian Randomization (MR) across large-scale genetic datasets from the Million Veteran Program, FinnGen, and UK Biobank. By mapping genetic variants associated with gene expression and protein abundance to 1,449 harmonized human phenotypes, we systematically identified potential adverse drug reactions (ADR). Our extensive MR analysis, encompassing 16,915 protein-coding genes, demonstrated the capacity to predict hundreds of known ADR for approved medications, with approximately 40% corroborated by FDA Adverse Event Reporting System (FAERS) data. Additionally, we found significant enrichment of identified gene-mechanism pairs in clinical trials terminated early due to safety concerns, highlighting the clinical utility of genetics-informed safety prediction. Notably, immune-related pathways were prominently associated with ADR, indicating particular sensitivity within immune modulation targets. Our comprehensive atlas, integrating genetic evidence with pharmacological mechanisms, provides a robust predictive framework for anticipating drug safety, potentially enhancing decision-making in drug development and pharmacovigilance. An interactive web interface allowing filtering by gene, phenotype, drug phase, and mechanism of action is available at https://shiny.parse-health.org/safety/.

## Author summary

Safety-related failures remain a substantial challenge in drug development, often emerging late in clinical trials or post-marketing. To address this, we developed a genomic-led framework that anticipates adverse drug reactions (ADRs) by leveraging Two-sample Mendelian Randomization (MR) across large-scale genetic datasets from the Million Veteran Program, UK Biobank, and FinnGen. Our approach systematically mapped genetic proxies for gene expression and protein abundance to 1,449 human phenotypes, generating an atlas of 8,495 gene–mechanism of action pairs linked to potential safety concerns. This resource recapitulates hundreds of known ADRs for approved drugs, with ~40% confirmed in FDA Adverse Event Reporting System data. Immune-related pathways were disproportionately associated with predicted ADRs, highlighting the sensitivity of immune modulation targets. We also demonstrate a liver-focused application that describes hepatotoxicity into mechanistic axes, improving interpretability and prediction. An interactive web visualization tool enables researchers to explore gene-level safety signals by drug phase and mechanism of action.

## Introduction

Safety-related issues are responsible for approximately 25% of new drug discovery program failures, and even among drugs that reach approval, unexpected adverse drug reactions (ADRs) often surface only after widespread clinical use [1,2]. The development of a framework to predict adverse drug reactions (ADR) could substantially increase the success rate of drug development programs as well as the identification and reporting of ADRs for novel and existing drugs [3]. In this context, even partially predictive models for ADRs are extremely valuable because they can alter the course of drug development, regulatory review, and post-marketing monitoring. For example, during early drug development, a partially predictive model could flag compounds at risk of hepatotoxicity, prompting additional toxicity assays or careful patient selection that could avert failures [4]. In regulatory review, such models could prioritize drugs for enhanced risk management plans, as seen with clozapine, where modest genetic predictors help guide monitoring strategies for rare but serious blood disorders [5]. Post-marketing surveillance systems could use model-derived risk scores to focus monitoring on patients at elevated genetic or clinical risk for ADRs. Even in drug repurposing, partially predictive models could identify patient subgroups where side effect profiles are more favorable, supporting broader and safer therapeutic applications.

A genetics-based strategy has been proposed aiding in the identification of potential drug targets for a number of human diseases. Recently, our group has created a large catalog of genetic risk factors for hundreds of human phenotypes and mapped those to potentially druggable targets using a Mendelian Randomization (MR) [6] approach. This method leveraged several publicly available data sources as genetic instruments of molecular exposures for gene transcripts or circulating proteins. Our initial analysis was able to identify new targets for hundreds of phenotypes, as well as different repurposing opportunities for previously approved drugs [6].

Here we propose to use a similar approach, adding the various mechanisms of actions for existing and to-be-developed drugs, and describe an atlas of drug targets that might lead to different types of ADR. First, we use MR at scale to list the potential safety concerns of targeting all protein-coding genes on 1,449 human phenotypes. We show that our approach is capable of recapitulating hundreds of known ADR for approved drugs. We provide publicly available results where researchers can, for any given protein-coding gene and mechanism of action, retrieve genetic signals that point towards the generation of potential ADR. One advantage of a genomic-led approach is that it does not rely on available selective compounds and as such can be applied early in the drug discovery program to anticipate safety liabilities.

## Results

### A catalog of potential adverse drug events

**Fig 1:**
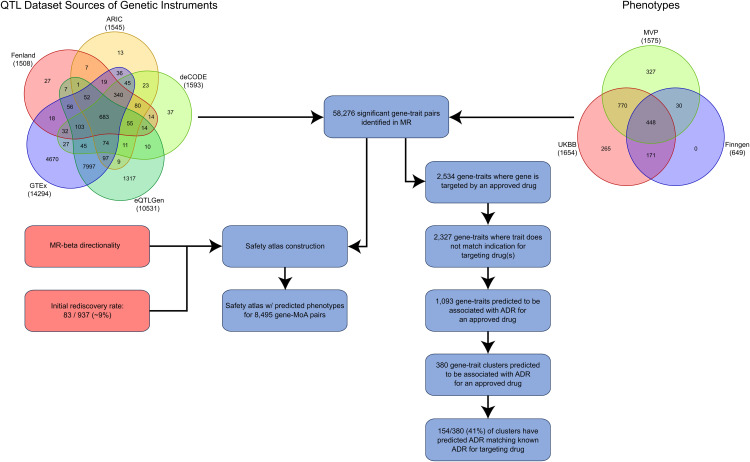
Flowchart of significant gene trait pairs and their corresponding safety events.

The two-sample MR filtering strategy and the analysis flowchart are represented in Fig 1. We started with 58,276 gene-trait pairs that passed the threshold for MR significance (p-value < 1.59x10-9). From these we mapped the predicted MoA that would lead to an adverse event (e.g., a positive or negative modulator). Briefly, we used the MR-beta directionality and the safety signal metrics (defined in Methods; Table A in S1 Table) to develop an atlas (Table A in S1 Table), in which, given a gene and a pharmacological action of a particular drug, we describe all of the genetically predicted safety concerns for that specific modulation of the target gene.

**Fig 2:**
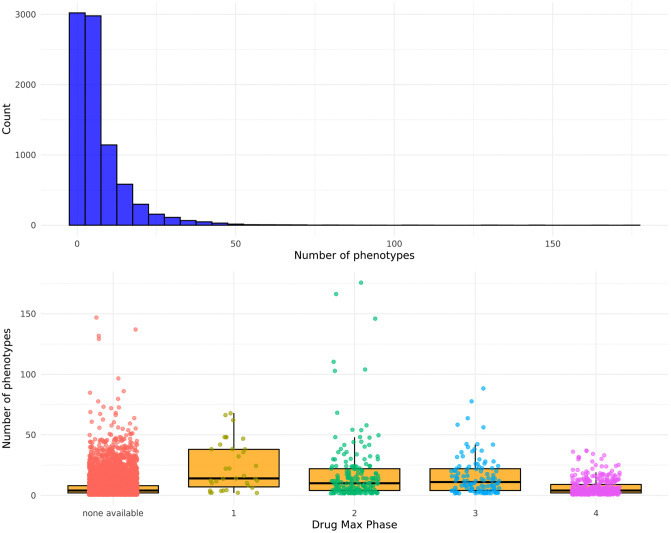
The mean number of phenotypes associated with each current development phase.

Our safety atlas describes predicted phenotypes for 8,495 unique gene-mechanisms of action (gene-MoA) pairs. The mean and median number of phenotypes for each unique gene-MoA pair was 7.2 and 4.0, respectively, indicating a right skew for the number of unique gene-MoA pairs. The gene HLA-DRB1 was the molecular mechanism predicted to be associated with the highest number of safety concerns (n = 176). As expected, regarding those gene-MoA for which there is a chemical compound in development as a treatment, the mean number of phenotypes associated with each gene-MoA was significantly associated with current development phase (Fig 2). Compounds in phase 1 had a median of 14 (mean of 24) predicted safety phenotypes, whereas for approved drugs (phase 4) the median number of associated phenotypes was 4 (mean 6.6), p-value = 4.1e-9.

To determine whether the clinical trait shared a causal variant with either the eQTL or pQTL source, we conducted colocalization analysis in all significant gene-traits identified as a potential safety concern [6]. Of the 58,276 gene-trait pairs in our atlas, 31,541 showed colocalization. These results can be found in Table B in S1 Table.

### Approximately 40% of genetically identified ADR for genes that target an approved drug have been previously reported in FAERS

**Fig 3:**
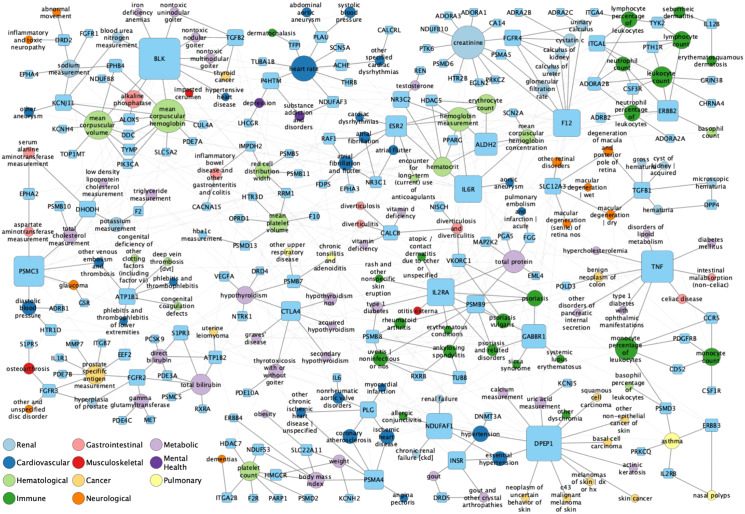
Network visualization of gene-trait pairs with known safety events from FAERS. Nodes represent both genes (square) and traits (circles) with node size scaled by degree, or number of connections. Color represents the high-level category of the trait. Communities were detected using Louvain detection algorithm. For this visualization, we reduce the width of inter-community edges to showcase the communities.

Genes that are the target of approved drugs provide another layer of confirmatory evidence for predicted adverse events because these drugs have stood the challenge of post-approval collection of physician and patient refereed adverse drug reactions (ADR). We have specifically analyzed our results in the context of genes and MoA for approved drugs. From the 747 genes targeted by approved drugs, according to the ChEMBL database version 34, there are 249 genes represented in our two-sample MR results. We used a four-step analysis to assess the agreement between an identified ADR and a known ADR as reported by FAERS. Initially, all significant gene-traits in which the gene is targeted by an approved drug were selected. This resulted in 2,534 significant gene-trait pairs (249 different genes and 422 different traits). Next, we excluded those gene-trait pairs in which the trait is an approved drug indication, resulting, after filtering, in 2,327 gene-trait pairs. These were then compared with respect to the MR-predicted directionality of effect (whether increasing or decreasing the exposure to the instrumented molecular phenotype is associated with decreasing or increasing risk of the phenotype). Finally, we contrasted that information with the known mechanism of action of the approved drug targeting the gene. This scheme was repeated for all selected gene-trait pairs. From the 2,327 considered gene-trait pairs our approach identified 1,093 that are predicted to be associated with an ADR for an approved drug. We then used the FAERS database and retrieved all known ADR for all approved drugs targeting the selected genes. This resulted in 205,805 potential ADRs listed in FAERS for the 650 different drugs targeting the 161 genes identified in this set of results. Since some of the 1,093 traits are very similar, such as “platelet count decreased” and “thrombocytopenia” (Table C in S1 Table), for each gene where several traits were related, we have manually clustered common traits to avoid double counting. This resulted in 380 unique gene-trait clusters where the gene is targeted by a known approved drug. We have systematically mapped all gene-trait clusters to all described ADRs for the approved drugs that target the gene. These gene-trait pairs with known ADRs are represented visually as a network graph in Fig 3. We were able to identify 154 (41%) positive ADR matches among the 380 unique gene-clusters associated with an approved drug. Severity was not significantly associated with FAERS mapping status, with severe ADRs (FAERS categories 3–4) showing slightly lower but non-significant odds of being mapped compared with non-severe events (OR = 0.72, 95% CI 0.45–1.16). In contrast, colocalization was significantly associated with FAERS mapping, as gene–phenotype pairs with positive colocalization had higher odds of being mapped in FAERS (OR = 1.62, 95% CI 1.06–2.46). (Table D in S1 Table). This result is similar to what we have previously described for the rediscovery of approved drug indications among a similar set of approved drugs and is associated with a 1.62-fold enrichment (p < 0.001) (that is, having MR evidence is associated with higher likelihood of seeing that phenotype listed as AE in FAERS). The fact that 40% of our predicted ADR for approved drugs have been, empirically, previously described for drugs targeting said genes, adds credence to our results.

### Genetically Identified ADRs have significantly higher odds of being identified by other sources of genetic information associated with the phenotype

**Fig 4:**
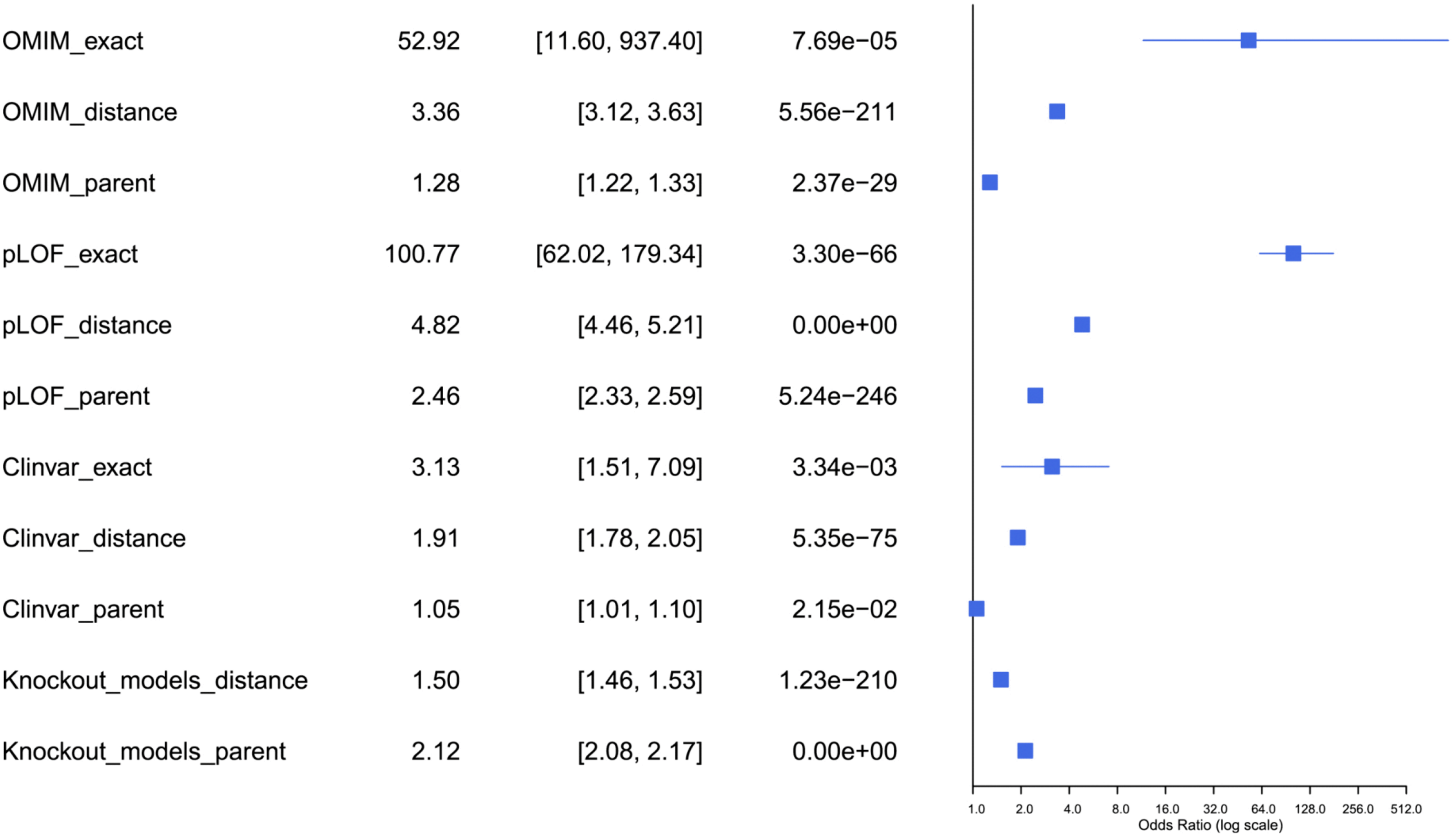
Enrichment of orthogonal sources in significant MR hits.

Additionally, we show an expected enrichment of the observed gene-trait pairs that constitutes a significant result from our atlas in relation to other sources of biological annotations, independently, providing support to our approach. We have mapped our safety gene-traits to other existing sources of biological annotations derived from genetic information. These were as follows: the existence of information linking the gene to the same trait in OMIM [7], the presence of gene variants causing the phenotype in ClinVar [8], the existence of a significant gene burden of damaging rare variants in the gene [9] and the presence of the phenotype, and the occurrence of the phenotype when the gene is targeted in a mouse model [10]. For all tested independent sources of biological support, gene-traits observed to be significant in our approach were also significantly enriched for a positive mapping by these other sources when compared to a set of gene-traits created by shuffling genes and associated phenotypes (Table E in S1 Table, Fig 4).

We did not observe any qualitative difference regarding the enrichment of the used biological annotations when comparing ADR events predicted to be derived from inhibiting the target gene against those derived from activating the target gene (S1 Fig).

### Genetic factors/patterns associated with genetically predicted ADRs

In order to further explore if we could identify predictive features of a significant gene-ADR MR result we have conducted exploratory analysis annotating our significant gene-ADR pairs for a number of potential predictors. Looking into gene-MoA pairs, we observed 4,142 that were associated with negative-modulators and 4,136 that were associated with positive-modulators (and 217 where both a negative-modulating and positive-modulating action would lead to an ADR). We observed a median number of 4 (mean 7.3) predicted ADRs for both negative-modulators and positive-modulators. In addition, no significant difference in the number of predicted ADR could be associated with the mechanism of action at any stage of drug development (S2 Fig). Taken together, these results suggest that genetically predicted ADR events are not more commonly associated with positive-modulators than with negative-modulators.

Next, we mapped all our genes from significant gene-trait pairs to gnomAD [11] conservation metrics (pLI and missense variants z-score). While we did not identify an association between the number of ADR phenotypes and pLI value (a conservation metric for LOF variants), we observed a significant association between the number of ADR phenotypes and the missense variants z-score (rho, 0.04, p-value = 0.0009).

Mapping all the genes from all significant gene-trait pairs to their associated GO [12,13] terms and then deconvoluting this information to understand what GO terms were most associated with the number of genetically predicted ADR events, we were able to initially identify 77 pathways whose modulation is predicted to be associated with a higher number of ADR phenotypes at an FDR-adjusted p-value < 0.0001 (Table F in S1 Table). The vast majority of observed GO terms associated with a higher number of genetically predicted ADR phenotypes were immune-related terms. Since several HLA genes were among the genes associated with the highest number of ADR phenotypes (among the top 8 genes were: HLA-DRB1, HLA-DQA2, HLA-DQB2, HLA-DQB1, HLA-DQA1, HLA-DRB5), we have conducted a sensitivity analysis removing the top 10 genes. Even after removing these genes, we observed 41 significantly enriched GO terms with a preponderance of immune-related pathways such as peptide-antigen binding, acute-phase response, cytokine activity, and positive regulation of T-cell-mediated cytotoxicity (Table G in S1 Table). Interestingly, the clear predominance of immune-related pathways being associated with a higher number of ADRs was observed both for positive modulation of identified targets and for negative modulation of identified targets (Table H in S1 Table). These results suggest that our approach has a high sensitivity to predict abnormalities in immune-related traits and that a significant proportion of our identified gene-traits can pleiotropically influence immune and inflammatory ADRs.

### Gene-mechanism of action pairs identified using MR information are significantly enriched in mechanisms of action that fail clinical trials due to safety issues

Finally, we observed a significant association between gene-MoA of action identified in our efforts and those used in a catalog of clinical trials in which early termination was due to increased safety or toxicity issues. From all drug combinations used in the 269 identified trials with early termination due to safety concerns, we identified 262 unique drugs used. We hypothesize that the molecular mechanism of each of these 262 drugs can potentially be a safety-related molecular mechanism. From these 262 drugs and their primary human gene targets (Table I in S1 Table), we identified 170 distinct gene-mechanism of action pairs on drugs used in failed clinical trials due to safety. Gene-mechanism of action pairs identified in our dataset of failed trials were 1.7-fold more commonly reported in our atlas of genetically mapped safety events than in non-significant gene-mechanism of action pairs (OR = 1.7, 95%CI 1.5 – 1.9, p-value 3.5e-13).

### A liver-focused phenotype panel resolves cholestatic and hepatocellular injury axes and helps to predict hepatotoxicity of known drugs

**Fig 5:**
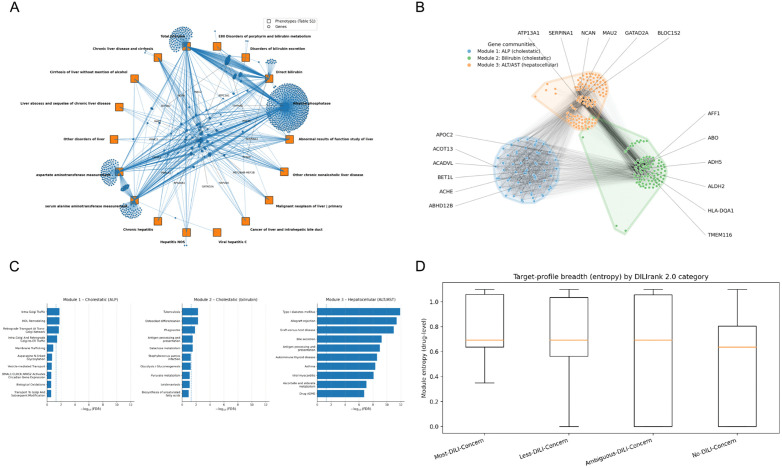
Network-based liver injury modules, drug mapping, and target-profile breadth across DILIrank 2.0 categories. **(A)** Gene–phenotype network. Bipartite network representation of genes and liver-related clinical phenotypes used to construct the initial atlas. Nodes represent genes or phenotypes, and edges denote reported gene–phenotype associations. No module assignments or enrichment information are shown, illustrating the unbiased structure of the input data prior to community detection. **(B)** Module-specific gene networks. Gene–gene networks corresponding to the three liver injury modules identified by community detection. Nodes are colored by module membership. Selected genes are labeled based on their inferred biological or clinical relevance to the dominant liver injury domain of each module, rather than by network centrality alone. **(C)** Pathway enrichment by module. Bar plots summarizing the most significantly enriched biological pathways for each module. Pathways are ranked by −log10(p-value) using precomputed enrichment statistics. Enrichment analyses were not re-run for figure generation. **(D)** Target-profile breadth across DILIrank 2.0 categories. Distribution of drug-level target-profile breadth, quantified as module entropy, stratified by DILIrank 2.0 categories (Most-DILI-Concern, Less-DILI-Concern, Ambiguous-DILI-Concern, and No-DILI-Concern). Entropy reflects the dispersion of a drug’s targets across liver injury modules, with higher values indicating broader, multi-module target engagement.

We performed a focused liver-toxicity analysis to illustrate how the current resource, by using a phenotype panel, rather than a single outcome definition, can decompose drug-induced liver injury (DILI) into interpretable mechanistic axes. Starting from the phenotypes currently available in the atlas, we identified 18 unique liver-related traits. To preserve clinical interpretability, each trait was assigned to one of seven liver-domain categories. We then retrieved all gene-trait associations identified through our approach for these 18 liver-related traits. This procedure was able to capture 964 unique genes associated with the selected traits (Table A in S2 Table). Evidence was concentrated in quantitative liver injury markers and clearly separated into cholestatic versus hepatocellular axes (Table B in S2 Table) (Fig 5, panel A).

### Gene–phenotype modularity across the 18-phenotype liver-injury panel identifies three major gene modules dominated by ALP, bilirubin, and ALT/AST signals

To identify coherent subsystems, we constructed a bipartite gene–phenotype graph within the liver subset and projected it to a gene–gene co-occurrence network, where edge weight corresponds to the number of shared liver phenotypes linking each pair of genes. We then applied modularity-based community detection to define gene modules supported by shared liver phenotype evidence (S3 Fig; Table C in S2 Table). In this liver-focused subset, three major modules emerged, each aligned with a distinct laboratory injury axis. Module 1 (458 genes) was dominated by the cholestatic marker domain anchored to alkaline phosphatase (ALP), with high-degree hub genes including HLA-B, SHBG, DDT, GSTT2, ARHGAP27, and OASL; Module 2 (257 genes) was dominated by the cholestatic marker domain anchored to bilirubin, with high-degree hub genes including ALDH2, TMEM116, HLA-DQA1, RNF5, ABO, and SLC35G2; Module 3 (249 genes) was dominated by the hepatocellular injury marker domain anchored to ALT/AST, with high-degree hub genes including MAU2, ATP13A1, NCAN, KRT8, IL1RN, and HP (Tables D through M in S2 Table provide detailed information on the community detection analysis results). Importantly, these modules provide a compact abstraction in which downstream biological interpretation can proceed within-module, while preserving the phenotype axis that anchors interpretability. It should be highlighted that module membership was inferred without manual curation beyond the liver phenotype selection and domain labeling.

### Module-wise pathway enrichment and pathway-level overlap

To determine whether the gene-level separation across pre-defined liver clinical domains also translated into pathway-level separation, we performed module-wise pathway enrichment (Enrichr [14–16]; Reactome 2022 [17], GO Biological Process 2023 [18,19], and KEGG 2021 Human [20–22]) using the gene members of each of the three major modules (Table L in S2 Table). We considered pathways significant at FDR q ≤ 0.05 and report complete enrichment results in Table M in S2 Table. Module 1 (n = 458 genes; primarily ALP/cholestasis) showed selective enrichment for vesicular and Golgi trafficking and lipid handling, including Intra-Golgi Traffic, HDL Remodeling, and retrograde Golgi transport processes (all FDR < 0.05 in Reactome). Module 2 (n = 257 genes; primarily bilirubin/cholestasis) was enriched for antigen processing and phagosome-related immune programs (Phagosome (KEGG) and Antigen processing and presentation (KEGG)) together with carbohydrate and alcohol metabolism signals (Galactose metabolism (KEGG) and Ethanol oxidation (GO:BP)). Module 3 (n = 249 genes; primarily ALT/AST hepatocellular injury) captured a broad immune–metabolic axis of liver injury, including Bile secretion (KEGG), xenobiotic disposition pathways (Drug ADME (Reactome) and Glucuronidation/ Phase II conjugation (Reactome)), oxidative metabolism (Biological oxidations (Reactome)), and immune checkpoint/interferon signaling (Interferon gamma signaling and PD-1 signaling (Reactome)), consistent with known roles of drug metabolism and immune-mediated inflammation in hepatotoxicity (Fig 5, panel C).

We then quantified pathway-level overlap across modules to contrast pathway-level sharing with the gene-level orthogonality observed across liver domains. Using exact term-name matching among significant pathways, only 6 pathways were enriched in more than one module (Table N in S2 Table), and all shared terms were restricted to Modules 2 and 3, dominated by antigen processing and presentation and phagosome-related terms. A similarity-based analysis of pathway labels (TF–IDF cosine similarity on term names; threshold 0.75) identified 8 high-similarity cross-module pathway pairs (Table O in S2 Table), again confined to immune/antigen presentation processes. Notably, no pathways were shared between Module 1 and either of the immune/metabolic modules, indicating that the lipid/vesicle-transport program captured by Module 1 is pathway-distinct, whereas Modules 2 and 3 converge on overlapping immune biology despite being partially gene-disjoint. Together, these results suggest that the atlas network supports both domain-aligned module structure at the gene level and that this reflects a more nuanced pattern at the pathway level in which orthogonality is preserved for some mechanisms (e.g., Golgi/HDL remodeling).

### External validation of phenotype specificity using LiverTox clinical injury patterns

We mapped all drugs targeting any of the genes associated with liver-related traits. 159 unique drugs mapped to the identified genes (65 approved (phase 4), 27 in module 1, 27 in module 2, and 8 in module 3). We then mapped atlas drug names to a corresponding LiverTox record when available and extracted two standardized fields: (i) the LiverTox likelihood score and (ii) the described clinical pattern of injury (Table P in S2 Table). We were able to map 41 approved drugs (31 after deduplicating compounds, 26 single-module and 5 multi-module). Interestingly, using this small number of approved drugs mapped to our genes, drugs whose targets spanned multiple gene modules appear to be more likely to have documented clinically apparent DILI, whereas drugs whose targets were confined to a single module were not linked to clinically apparent acute liver injury (12 of the 26 single-module drugs were labeled as hepatotoxic by LiverTox, and 4 out of the 5 multi-module drugs were labeled hepatotoxic).

### Network expansion maintains module specificity and provides a tool for predicting hepatotoxicity

The previous observation using LiverTox data suggests that cross-module target profiles may mark broader hepatotoxic liability. To test whether cross-module target profiles correspond to broader hepatotoxic liability, we first expanded our 3 modules using STRING [23] (STRING combined score > 0.8). This expanded the number of unique genes from 964 to 6,934 in the expanded modules (Table Q in S2 Table). We next used the FDA Liver Toxicity Knowledge Base DILIrank 2.0 dataset [24], which assigns 1,336 FDA-approved drugs to four ordinal DILI-concern categories based on FDA labeling and a literature review and mapped 1,032 of 3,366 module-mapped drugs to DILIrank 2.0 (Table R in S2 Table). For the primary test, we dichotomized DILIrank into DILI-positive (Most-DILI-Concern or Less-DILI-Concern) versus DILI-negative (No-DILI-Concern), excluding Ambiguous-DILI-Concern from the binary analysis. Under this definition, multi-module drugs in the expanded dataset had significantly higher odds of DILI-positive labeling than single-module drugs (odds ratio 2.55, 95% CI 1.84–3.55; Fisher’s exact p = 2.84e-08; counts: multi-module yes/no = 396/161, single-module yes/no = 99/103). Across the full four-class labeling, multi-module drugs were enriched for higher-concern categories and showed greater target-profile breadth (entropy) relative to No-DILI-Concern drugs (Fig 5 panel D).

Because drugs that map to multiple modules also tend to have more mapped targets, we evaluated whether the DILIrank association reflects module breadth or simply the number of mapped target genes. We fit logistic regression models for DILI-positive status (Most/Less vs No; Ambiguous excluded) including both number of modules and target gene count as covariates. In these models, the estimated effect of number of modules remained positive after adjustment for gene count, indicating that cross-module targeting carries information beyond total target count (Table S in S2 Table).

To directly test the question of whether mapping to two targets in different modules differs from mapping to two targets within the same module, we next stratified drugs by their total number of mapped target genes and compared DILI-positive rates for multi-module versus single-module drugs within each stratum (Table T in S2 Table). Among drugs with exactly two mapped targets, cross-module drugs showed higher odds of DILI-positive labeling than same-module drugs (OR 3.08, 95%CI1.37 – 6.91, p-value 0.008), supporting the interpretation that module breadth captures clinically relevant pleiotropy not fully explained by target count alone. All processes regarding the hepatotoxicity vignette are described in the supplementary file S1 Text.

## Discussion

This study presents a systematic approach to leveraging Mendelian randomization (MR) and genetic evidence for identifying adverse drug reactions (ADR) associated with gene-target modulations. By integrating data on gene-trait relationships with pharmacological mechanisms, we developed a comprehensive safety atlas that predicts potential adverse outcomes of therapeutic interventions. This atlas represents a significant advancement in drug safety research, contributing to both drug development and post-market safety assessments.

Our findings align with growing evidence on the utility of human genetics in improving drug safety predictions [3,25]. Recent reviews [3,25–27] highlight the increasing use of genetic data to predict safety liabilities, especially for novel drug targets lacking robust animal model validation or translational pharmacology data. By identifying 8,495 unique gene-mechanism of action (gene-MoA) pairs and demonstrating their association with varying numbers of phenotypic outcomes, our approach corroborates the concept that genetic proxies can simulate pharmacological modulation effects of target genes.

We demonstrated that gene-trait pairs identified in this atlas are significantly enriched in annotations from databases such as OMIM, ClinVar, and functional mutation burden studies. These findings extend previous work on integrating genomic annotations into drug development pipelines. The enrichment highlights the translational value of combining MR with external genetic datasets to corroborate safety predictions.

### Concordance with existing pharmacovigilance data

The observed 40% overlap between genetically predicted ADRs and those reported in the FAERS database for approved drugs underscores the validity of using MR-based predictions as a pharmacovigilance tool. The significant enrichment of ADR matches (p < 0.001) suggests that genetically derived insights can complement existing data sources, enhancing the accuracy of ADR identification. This result is consistent with previous studies demonstrating the concordance of genetically informed safety signals with clinical observations in post-market surveillance [25,28].

Despite this, it should be noted that, solely, the identification of a significant gene-trait has a low positive predictive value to help predict an ADR of an approved drug (60% of suggested gene-trait pairs observed for genes targeted by approved drugs could not be mapped to any ADR in FAERS for these drugs). This could be due to several concomitant facts. For example, MR results can be biased by horizontal pleiotropy due to the potential non-specific nature of genetic instruments; long-term genetic exposures are inherently different than the acute exposure to a drug (genetic exposures are present since inception and may not necessarily recapitulate the complex pharmacology of a drug exposure); the effect sizes associated with the mapped gene-traits might be small and not surpass the needed threshold to characterize a clinical ADR.

### Pathways and mechanisms associated with ADRs

A key observation of this study is the disproportionate representation of immune-related pathways among gene-trait pairs associated with a higher number of genetically predicted ADRs. Pathway analysis revealed significant enrichment for terms such as “peptide-antigen binding” and “cytokine activity,” consistent with earlier findings linking immune modulation to adverse events. Even after sensitivity analyses excluding the top 10 genes, immune pathways remained prominent, reinforcing the hypothesis that genetic variation in immune-related genes frequently underlies ADR risks. Our finding that inhibition of HLA-DRB1 is associated with the highest number of safety concerns (n = 176) parallels known clinical challenges with immune-targeting therapies. This observation aligns with prior reports that genetic variants in immune-related loci are strong predictors of therapy-related adverse events, particularly for checkpoint inhibitors. From a practical perspective these findings also emphasize the value of scalable MR analysis in continuous traits that can serve as good proxies of immune-related adverse events, such as lymphocyte counts.

### Clinical relevance and future applications

Our results emphasize the utility of MR as a predictive tool for drug discovery. The observed association between gene-moa pairs and clinical trial terminations due to safety concerns (OR = 1.7, 95% CI: 1.5–1.9, p = 3.5e-13) suggests that genetically-informed safety predictions can guide early-stage decision-making in drug development. This is particularly relevant given the high attrition rates in clinical trials attributed to unanticipated safety issues [3]. While efficacy remains the leading cause of clinical trial failures, accounting for approximately 57% of Phase II and 45% of Phase III failures, safety concerns are also a significant contributor, responsible for about 17% to 20% of Phase III failures. Conversely, in preclinical stages, safety issues are more prominent, with toxicity being a primary reason for candidate attrition [29]. Integrating human genetic evidence early in the drug development process can enhance the prediction of both efficacy and safety outcomes. To further guide decision making, we have added the severity categories (see S2 Text) to the safety atlas which can be found in Table J in S1 Table.

Interestingly, our initial hypothesis was that positive-modulators would be more frequently associated with ADRs. In other words, that downregulating a gene (decreasing its expression or function) is generally more tolerated than upregulating it (increasing its expression or activity). Physiological systems often feature parallel pathways that can mitigate the effects of partial loss-of-function mutations, providing a level of resilience to negative modulation. However, our findings revealed a comparable distribution of ADRs across mechanisms that increased or decreased the expression of genes, suggesting that this compensatory capacity does not translate into a reduced safety burden for negative-modulators.

### Illustrative use of the resource: resolving liver injury axes and improving drug hepatotoxicity prediction

The liver-focused vignette presented here illustrates how the resource we make available can be used to move beyond single-outcome definitions of adverse drug reactions and instead decompose organ toxicity into mechanistically interpretable axes. By assembling a focused panel of clinically relevant liver phenotypes and integrating them with genetically informed gene–phenotype associations, we show that complex drug-induced liver injury (DILI) signals can be resolved into distinct cholestatic and hepatocellular dimensions. This use case demonstrates how the resource enables hypothesis-driven exploration of adverse events while preserving clinical interpretability and mechanistic granularity.

A key feature of this vignette is the semi-supervised analytical strategy underlying module identification. Rather than imposing predefined pathways or relying solely on unsupervised clustering of molecular networks, module structure emerged from shared genetic evidence across clinically defined phenotypes. This approach led to the identification of three major gene modules anchored to alkaline phosphatase, bilirubin, and ALT/AST signals, respectively. Importantly, these modules were inferred without manual gene curation beyond phenotype selection and labeling, highlighting how weak supervision at the phenotype level can guide biologically meaningful structure discovery in gene networks. Similar strategies have been proposed in other contexts, including phenotype-driven network stratification and genetically informed pathway discovery for elucidating disease mechanisms and supporting drug efficacy, but these approaches have rarely been extended to systematic modeling of adverse drug reactions or genetically informed drug safety prediction [30–32].

The resulting module structure proved additive for predicting hepatotoxicity. Drugs whose targets spanned multiple modules showed a consistently higher likelihood of clinically apparent liver injury across independent external resources, including LiverTox and DILIrank 2.0. This association persisted after accounting for the total number of target genes, indicating that cross-module targeting captures information beyond simple target pleiotropy. These findings are consistent with prior observations that polypharmacology and pathway pleiotropy contribute to safety liabilities [33,34].

More broadly, these results support the hypothesis that monitoring discrete biological programs derived from gene networks anchored in clinical and genetic evidence can improve the sensitivity of drug safety prediction algorithms. Existing approaches to DILI prediction have leveraged chemical structure [35], in vitro toxicity assays [36], transcriptomic signatures [37], or post-marketing surveillance data [38], each with well-recognized limitations. Recent efforts incorporating genetic constraint metrics, Mendelian randomization, and pathway-level annotations have begun to address these gaps, but often lack an explicit framework for integrating multiple clinical phenotypes within an organ system. Our results suggest that organizing genetic evidence into phenotype-informed modules provides a natural intermediate representation that bridges molecular mechanism and clinical outcome.

Fostamatinib provides a concrete example of how this resource can nominate drugs with a high a priori probability of hepatotoxic liability that are not highlighted by existing reference datasets. In the STRING-expanded analysis, fostamatinib mapped to all three liver injury modules and exhibited a high, near-maximal module entropy, indicating broad engagement of gene networks spanning cholestatic and hepatocellular pathways. Based on the associations observed across the expanded drug set, this cross-module target profile would predict an elevated likelihood of hepatotoxicity relative to drugs confined to a single module. Notably, however, fostamatinib is not categorized in DILIrank 2.0 and does not carry a strong or specific hepatotoxicity annotation in LiverTox. Nonetheless, Fostamatinib has documented hepatic safety signals primarily characterized by reversible elevations in ALT/AST (including values >3× ULN in a subset of treated patients), prompting routine LFT monitoring in regulatory labeling [39,40].

### Limitations and future directions

While our findings demonstrate the utility of Mendelian Randomization (MR) as a predictive tool for drug discovery and highlight the relationship between genetically informed safety signals and clinical trial terminations, several limitations must be acknowledged. The reliance on genetic proxies to infer pharmacological modulation may not capture all nuances of drug action, particularly for polypharmacological agents. For example, our analysis is restricted to protein-coding genes and therefore does not capture regulatory or non-coding RNA mechanisms, which may also play important roles in drug response and toxicity. Additionally, while the observed enrichments provide strong evidence of validity, functional validation in cellular or animal models remains critical for translating these findings into actionable insights. Future research should explore integrating secondary pharmacology panels and gene expression perturbation data to refine ADR predictions further. Resources such as PharmGWAS [41], which systematically combines genetic signatures with drug perturbation profiles, offer promising avenues for expanding the scope of safety assessments.

Although the adverse event data curated by Open Targets from the FDA Adverse Event Reporting System (FAERS) was cleaned and filtered to improve reliability, inherent challenges remain. The FAERS database relies on spontaneous reporting, and its effective population size (that is, the total number of exposed individuals) is unknown, which complicates estimates of absolute or relative risk. Furthermore, despite data cleaning, adverse events that resemble the original indication for which the drug was prescribed often persist in the dataset, making it difficult to distinguish between disease progression, off-target drug effects, or true adverse drug reactions (ADR). These factors may introduce residual confounding and misclassification that could bias our interpretation of gene–mechanism–ADR relationships. Future efforts to integrate pharmacovigilance data with well-annotated exposure denominators, detailed indication information, and prospective validation frameworks will be critical for refining genetically informed safety predictions and improving their translational relevance.

It should be acknowledged that our analysis was restricted to individuals of European ancestry. This choice was driven by methodological considerations, including the need to satisfy linkage disequilibrium assumptions underlying two-sample Mendelian randomization and to ensure harmonized LD structure between exposure and outcome datasets. However, adverse drug reaction (ADR) risk is known to vary across populations due to differences in allele frequencies, LD patterns, genetic background, and environmental context. As such, the results presented here should not be interpreted as estimates of ADR incidence or relative risk in specific ancestry groups. Rather, our framework is intended as a target- and mechanism-level screening tool to flag potential safety liabilities during drug discovery and early development, independent of population-specific risk magnitudes. Importantly, applying the same approach in non-European ancestry cohorts, once sufficiently powered molecular QTL and phenotype resources become available, may uncover additional gene–trait associations and safety signals that are not detectable in European populations alone. Expanding genetically informed safety prediction across ancestries therefore represents a critical future direction.

ADRs are often classified as predictable, mechanism-based (Type A) or idiosyncratic (Type B). By leveraging germline instruments for target gene expression and protein abundance, our MR screen is inherently tuned to predictable, on-target and pathway-mediated liabilities that would generalize across chemotypes acting on the same target. We additionally observe strong enrichment of immune-related pathways, indicating that host-genetic susceptibility to certain idiosyncratic events can be captured. Nonetheless, compound-specific idiosyncrasy (for example, reactions driven by reactive metabolites or specific HLA–drug interactions) typically requires integration of drug-level information and is not comprehensively addressed by our design

An additional limitation lies in the use and interpretation of clinical trial outcomes. Publicly available trial registries and databases often lack detailed, standardized reporting on the specific reasons for trial failure, particularly when trials are terminated early or fail to meet primary endpoints. Although we focused on trials explicitly classified as terminated for “safety” reasons, classifications can be imprecise or incomplete, with categories such as “business decision” or “strategic realignment” potentially masking underlying safety or efficacy concerns. Moreover, sponsors may underreport unfavorable outcomes to protect proprietary interests, and adverse events leading to discontinuation are not always uniformly adjudicated across different studies. In many cases, failure is multifactorial, with intertwined contributions from efficacy, safety, operational challenges, and commercial factors, making it difficult to assign a single causal attribution. These ambiguities limit the resolution with which we can validate genetically informed safety predictions against trial outcomes and underscore the need for enhanced transparency and structured post-hoc analyses of trial terminations.

## Materials and methods

### Experimental design

GWAS summary statistics were obtained from the Million Veteran Program [42] (MVP), FinnGen [43] R.10, and the UK Biobank [44,45] (UKBB). From UKBB and MVP, we included 1,556 unique Phecodes, 72 biomarkers, 74 questionnaire data, and 6 clinical variables. A total of 1,449 traits were harmonized between datasets and meta-analyzed. For meta-analyzed traits, we performed fixed effects inverse-variance weighted meta-analysis using METAL [46]. For traits where harmonization was not possible (MVP, n = 327; UKBB, n = 235), we used summary statistics from individual studies. FinnGen on its own was not used. Certain traits with no clear safety concern (for example height) were removed from consideration (n = 562) leaving a total of 1,449 traits. Given that the MR framework relies on the expectation that linkage disequilibrium (LD) is the same among genetic studies measuring the association among exposures and outcomes, this study includes samples of European ancestry only. Additional information regarding phenotype harmonization and meta-analysis can be found in “Leveraging Large-Scale Biobanks for Therapeutic Target Discovery” [47].

### Instruments for MR

We have used 5 different sources of genetic instruments in the present work. Below we describe the instrument selection criteria for each source.

#### GTEx version 8.

Independent cis-eQTLs were identified per gene by performing up to 5 conditional analyses in regions (+/-1 Mb from the transcription start site [TSS] of each gene) using GTEx v8 [48] individual-level data, additionally iteratively adjusting for the top associated variant if there exists an association reaching a p-value of 1e-4. The primary signal was unconditional for other variants in the region. To identify the independent signals, we considered primary and conditional associations passing a p-value < 5e-8. We then extracted estimates of effect size, corresponding modeled allele, and standard errors from the unconditional association to use in the next steps of MR. This approach was taken for each available GTEx tissue. We used 120,303 genetic markers instrumenting 24,116 genes.

#### eQTLGen.

Summary statistics files were downloaded from eQTLGen [49] https://eqtlgen.org/cis-eqtls.html,

Since only Z-scores and p-values, but not the betas, are listed, we have computed the beta and SE from the following formula: Beta = z/ sqrt(2p(1 − p)(n + z^2)); SE = 1/ sqrt(2p(1 − p)(n + z^2)), after downloading the MAF file provided here: https://molgenis26.gcc.rug.nl/downloads/eqtlgen/cis-eqtl/2018-07-18_SNP_AF_for_AlleleB_combined_allele_counts_and_MAF_pos_added.txt.gz.

The summary statistics reported SNP-gene associations (<1Mb from the center of the gene and tested in at least 2 cohorts) across 19,250 genes (17,114 in common between GTEx and eQTLGen). We defined instruments using the smallest p-value per gene. We used liftOver to convert SNPs from GRCh37 to GRCh38 genome build. There were 558 SNPs in GRCh37 that could not be converted and were dropped from our analysis. We used 10,669 markers instrumenting 11,175 genes [50].

#### deCODE.

We downloaded the published GWAS of SOMAscan v4 in 35,000 individuals of European ancestry for 4,907 aptamers from deCODE [51] (https://www.decode.com/summarydata/). We used 4,775 genetic markers instrumenting 1,624 genes.

#### Fenland.

We obtained pQTLs directly from the Fenland study [52], a genome-proteome-wide association study among 10,708 participants of European-descent conducted using 10.2 million genetic variants and plasma abundances of 4,775 distinct protein targets (proteins targeted by a least one aptamer) measured using the SOMAscan V4 assay on 4,979 aptamers (4,775 unique protein targets). The unconditional summary statistics from this study are available in an open resource platform (www.omicscience.org). We used 2,881 genetic markers instrumenting 1,510 genes.

#### ARIC.

We downloaded the published cis-pQTL GWAS from the Atherosclerosis Risk in Communities (ARIC) study [53] (http://nilanjanchatterjeelab.org/pwas/), which contains SOMAscan v4 on 4,657 plasma proteins measured in 7,213 European American individuals. SOMAmers that mapped to multiple gene targets, lacked a position record in the BioMart database for the target protein-coding gene, or lacked any SNPs in the cis region were excluded from further analysis.

The cis-region is defined as ±500 kb of the TSS of the target protein-coding gene in the cis-pQTL analysis. A total of 2,004 significant SOMAmers in the original study were therefore identified as having had at leastone cis-pQTL (FDR < 5%) near the gene of the putative protein. We used unconditional estimates from this list of 2,004 cis-pQTLs from the original study. We used 1,612 genetic markers instrumenting 1,594 genes.

### Mendelian randomization (MR)

Two-sample Mendelian Randomization (MR) [54] of each of 16,915 protein-coding genes was performed for all phenotypes using instruments, separately, from five sources of eQTLs and pQTLs. The datasets used for instruments provided summary statistics for the unconditional primary association. For each of the datasets described above, we used the instruments identified by the authors. We extracted the corresponding effect size and standard errors from the unconditional association to use in MR. In order to determine the correct ordering of alleles between the datasets we utilized the harmonise_data() function from the TwoSampleMR package in R. We used the Wald Ratio for instruments with one genetic variant and inverse-variance weighted MR for instruments with multiple genetic variants. We additionally performed MR-Egger for proteins/expression with three or more instruments to be used as a sensitivity analysis. We tested for heterogeneity across variant-level MR estimates, using the Cochrane Q method (mr_heterogeneity option in TwoSampleMR package) and the MR-Egger intercept.

We define genes with significant MR as genes using a Bonferroni correction with p-value ≤ 1.6e-9 for any MR test. This value was obtained by dividing 0.05 by the number of unique gene-trait pairs tested in our study (31,525,236 unique gene-traits initially tested). If a gene was significant in multiple QTL sources, we required the directionality of the beta values to be concordant, otherwise the result was excluded from our list of selected results. If a gene passed in both eQTL and pQTL sources, we only considered the pQTL result. These are publicly available and can be downloaded at the Department of Veterans Affairs Centralized Interactive Phenomics Resource (CIPHER) web portal (https://phenomics.va.ornl.gov/) or at https://osf.io/g8hjz/overview [55].

### Inferring direction of pharmacological modulation from mendelian randomization

To connect Mendelian randomization (MR) results with pharmacological mechanisms of action, we inferred the direction of target modulation from the sign of the MR effect. MR estimates the causal effect of a genetically proxied molecular exposure, gene expression or protein abundance, on a phenotype. A positive MR effect (β_MR_ > 0) indicates that increased expression or abundance of the target gene is associated with increased risk or level of the phenotype, whereas a negative effect (β_MR_ < 0) indicates a protective association. We therefore define positive modulation as any intervention expected to increase target activity or abundance and negative modulation as any intervention expected to decrease it. Under this framework, when β_MR_ > 0, positive modulation is predicted to increase risk and negative modulation to be protective; when β_MR_ < 0, the inverse applies.

This classification is based on directional concordance between genetic and pharmacological perturbation rather than drug-class specific terminology. We use “positive modulator” and “negative modulator” as umbrella terms to describe genetically inferred direction of target modulation, while retaining standard pharmacological labels (e.g., agonist/antagonist, inhibitor/activator) when referring to curated drug annotations. This approach assumes that genetically driven differences in expression or abundance approximate sustained on-target modulation and primarily capture pathway-mediated safety liabilities shared across compounds acting on the same target, rather than compound-specific or off-target toxicities. Within these assumptions, this strategy enables systematic integration of human genetic evidence into target-level safety assessment.

Finally, we have generated a curated list of phenotypic directions effects that we would consider as a safety signal or concern. For example, for a phenotype such as hemoglobin levels we considered a decrease in hemoglobin levels as a safety concern, but not an increase in hemoglobin levels. All predefined safety directions are available in Table A in S1 Table. Taking together the predicted genetic modulation of the target, the function of this modulation and an increased or decreased risk of the phenotype and the assumed safety signal, we were able to map all significant MR results to potential safety concerns bridging gene or protein expression directionality to an increase or decrease in phenotype odds. It is important to note that our list of safety signals is subjective and, although not disputable for the vast majority of phenotypes, new or modified directionalities might also be considered a safety concern.

### Colocalization analysis

For genes with a significant MR result, we performed colocalization between the outcome GWAS (or meta-analysis) and the cis-variants available from the QTL sources passing MR for that gene-trait using the coloc package in R. Marginal (unadjusted) eQTL/pQTL results and unconditional results on each of the instruments used in the MR were used. For GTEx instruments identified by conditional associations, since the full associations were available to us, the conditional results were used. We used variants with MAF > 1% and a ±250-kb window around each of the instruments. We defined colocalization as having posterior probability for hypothesis 4 (PP.H4) > 0.5 (the probability of a shared causal variant) for at least one IV. Prior probabilities were set to default values.

### Mapping gene results to existing drug data

We extracted drug information for protein targets from ChEMBL (v34) [56]. For all protein targets, we acquired Ensembl IDs when available using UniProt’s REST API [57]. For each drug where the information was available in ChEMBL, we identified all indications for those drugs and the clinical phase for that indication. We distinguish between genetic modulation direction inferred by MR (positive vs negative modulation of target *activity/abundance*) and pharmacological action types drawn from curated drug databases. We therefore use “positive modulator” and “negative modulator” as umbrella terms for genetics, and reserve class-specific pharmacology terms (e.g., agonist/antagonist for receptors; inhibitor/activator for enzymes; blocker/opener for ion channels; inhibitor/substrate/modulator for transporters) when we reference approved or investigational drugs. Drug information can be found in Table K in S1 Table.

### Enrichment of functional and genetic annotations among safety-associated genes

We also evaluated whether genes implicated by the safety atlas exhibit enrichment for genetic constraint and functional pathway membership. Gene-level loss-of-function constraint metrics were obtained from gnomAD v2.1.1 and integrated with safety-associated genes to assess intolerance to disruptive variation. Functional enrichment was evaluated using Gene Ontology (GO) annotations retrieved via biomaRt from the Ensembl human gene database. Genes were expanded into a binary gene–GO matrix, and associations between GO term membership and the number of associated safety phenotypes per gene were tested using negative binomial regression, accounting for overdispersion in phenotype counts. One-sided p-values were computed and adjusted for multiple testing using the Benjamini–Hochberg procedure. Significant GO terms were annotated using Ensembl and GO.db resources. Sensitivity analyses excluding the top ten most pleiotropic genes yielded consistent enrichment patterns, indicating that results were not driven by a small subset of genes. Stratified analyses by predicted mechanism of action (positive versus negative modulation) revealed broadly similar enrichment profiles, with both agonist- and inhibitor-associated genes showing significant functional convergence.

### Adverse drug event data

We used adverse event data reported in the FDA Adverse Event Report System (FAERS) (n = 36,653). This data was acquired from Open Targets [58] who processed the FAERS data. Briefly, Open Targets included only reports submitted by healthcare professionals, excluded reports where the outcome was death, and removed events deemed uninformative based on a manually curated exclusion list. We only used drug-ADR that reached statistical significance using the Open Targets approach.

FAERS adverse event annotations were integrated with the mapped gene–phenotype dataset by harmonizing adverse event terms and appending standardized FAERS severity classifications to each record (Table L in S1 Table). We then assessed whether FAERS mapping status differed by severity or colocalization using contingency table analyses. Odds ratios and 95% confidence intervals were estimated from 2 × 2 tables, with statistical significance evaluated using Fisher’s exact tests and chi-square tests of independence where appropriate.

### Integration of orthogonal genetic annotations

To evaluate the biological support for gene–trait associations identified by the safety atlas, we integrated multiple independent sources of genetic and experimental evidence. Significant gene–trait pairs were combined with a background set of non-significant pairs generated by shuffling genes and phenotypes while preserving dataset structure. Each gene–trait pair was represented by a unified gene–phenotype identifier. Orthogonal annotations were derived from external resources and included evidence from OMIM, ClinVar, gene-level burdens of damaging rare variants, and mouse knockout models. Annotations were captured at varying levels of phenotypic resolution (exact, parent, and distance-based mappings) and merged by exact gene–phenotype matching. Enrichment was assessed by comparing the frequency of positive annotations among significant gene–trait pairs against the non-significant background using contingency table–based statistical tests. Analyses were additionally stratified by predicted mechanism of action to evaluate consistency across target activation and inhibition.

### Clinical trials with early termination due to increased safety concerns

We determined clinical trials that were terminated due to increased safety concerns by systematically filtering trial data. Specifically, we used a previously assembled database of clinical trials (n = 17614) [59]. First, we identified trials flagged as “Terminated,” “Withdrawn,” or “Suspended” in the <overall_status> field to capture all studies that ended earlier than planned. We then reviewed the < why_stopped> field for each record to identify those citing “safety” or “adverse events” as a primary reason for early termination. After excluding duplicates and records in which the stated reason for termination was ambiguous (e.g., “business decision,” “futility,” or “slow enrollment”), 269 trials remained that met our criteria for termination early due to safety concerns. These final 269 trials were included in the subsequent analyses (Table M in S1 Table). The raw trial data, which included various drug and placebo combinations, was parsed to extract unique drug names for each trial arm. A custom R function was employed to clean and parse the drug names from the trial data, ensuring that only drugs with human molecular targets were included, while placebo and other non-drug interventions were excluded. The identified drugs were then mapped to their respective gene-level molecular targets using the existing pharmacological databases DrugBank [60] and PubChem [61]. This mapping allowed us to analyze the molecular mechanisms associated with the drugs used in these trials and their potential contributions to the observed safety concerns. The final dataset of drug-target relationships was compiled and made available for further analysis and integration with other clinical trial data.

### Statistical analysis

We compared the median number of identified gene-MoA associations by drug approval phase using a Wilcoxon rank sum test due to the skewed distribution of this variable. We compared the median number of phenotypes associated with negative modulators versus positive modulators using a Kruskal_Wallis rank sum test. The association between the number of ADR and missense or LOF constrain scores was evaluated through a Spearman correlation test.

## Supporting information

S1 Fig Forest plots of both agonists (A) and inhibitors (B). (DOCX)

S2 Fig Mean number of phenotypes by both mechanism of action and drug phase. (DOCX)

S3 Fig Number of genes found in each community stratified by disease domains. (DOCX)

S1 Table Supplementray Tables A–M. **Table A.** Gene-Trait Safety Atlas. **Table B.** Colocalization results. **Table C.** Clustering of ADRs by gene. **Table D.** FAERS mappings by positive colocalization. **Table E.** Enrichment of orthogonal sources in significant MR hits. **Table F.** Pathways whose modulation is predicted to be associated with a higher number of ADR phenotypes. **Table G.** Significant GO Terms with immune-related pathways. **Table H.** Significant GO Terms stratified by mechanism of action. **Table I.** Drugs, their primary human target and corresponding mechanism of action for drugs whose trials were terminated early due to safety concerns. **Table J.** Safety Atlas with severity variables. **Table K.** Drug-target information for Chembl 34. **Table L.** Classification of FAERs events by disease severity. **Table M.** Trials terminated early due to safety concern.
(XLSX)

S2 Table Supplementray Tables A–T. **Table A.** Liver phenotype panel. **Table B.** Domain summary. **Table C.** Major modules of the network graph. **Table D.** Domain support by gene communities. **Table E.** Domain support metrics. **Table F.** Domain enrichment hypergeom. **Table G.** Multidomain gene summary. **Table H.** Multidomain gene list. **Table I.** Domain gene overlap jaccard hypergeom. **Table J.** Domain edge mixing enrichment ratio. **Table K.** Domain edge mixing enrichment ratio primary domains. **Table L.** Module gene list. **Table M.** Module gene list summary. **Table N.** Shared enriched pathways across modules. **Table O.** Similar enriched pathway pairs. **Table P.** Livertox mapping with mapped drugs. **Table Q.** STRING0p8 expanded module gene list. **Table R.** Dilirank2 mapping to STRING0p8 module drugs. **Table S.** Modules vs gene count based on logistic regression. **Table T.** Stratified DILIrank2 by gene count and module multiplicity.
(XLSX)

S1 Text File containing a more in-depth description of the methods related to the liver-focused vignette. (DOCX)

S2 Text File containing a more in-depth description of the methods. (DOCX)

S3 Text A full acknowledgement for the Million Veteran Program. (PDF)
