# Supplementary figures and images for: A genomic-led strategy to anticipate drug safety effects
Source: PLoS Genet. 2026 Jul 16;22(7):e1012211. doi: 10.1371/journal.pgen.1012211 (PMC13375020; doi:10.1371/journal.pgen.1012211)

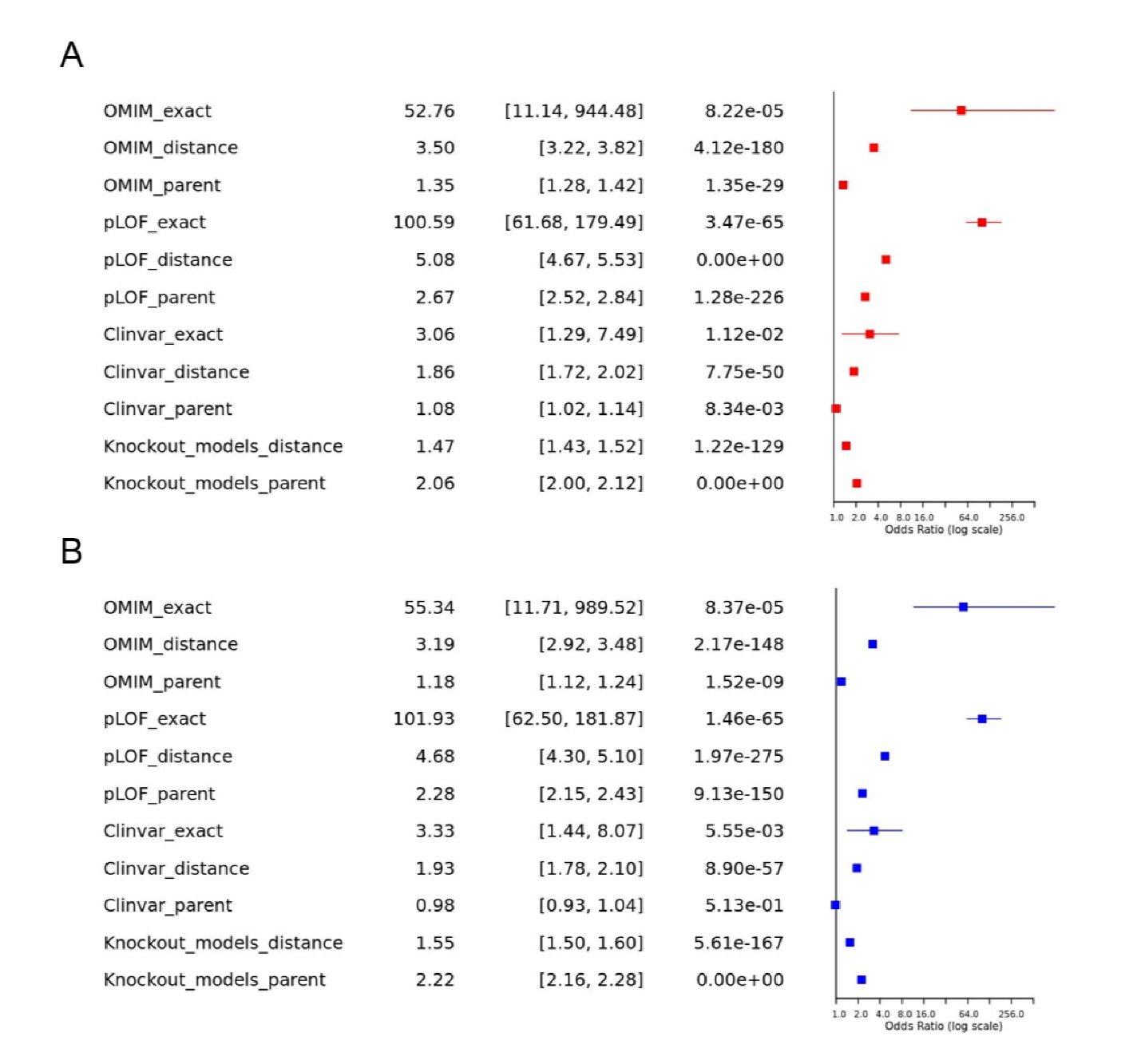


S1 Fig – Forest plots of both agonists (A) and inhibitors (B))

Supplement: S1 Fig — (DOCX) [file pgen.1012211.s001.docx]

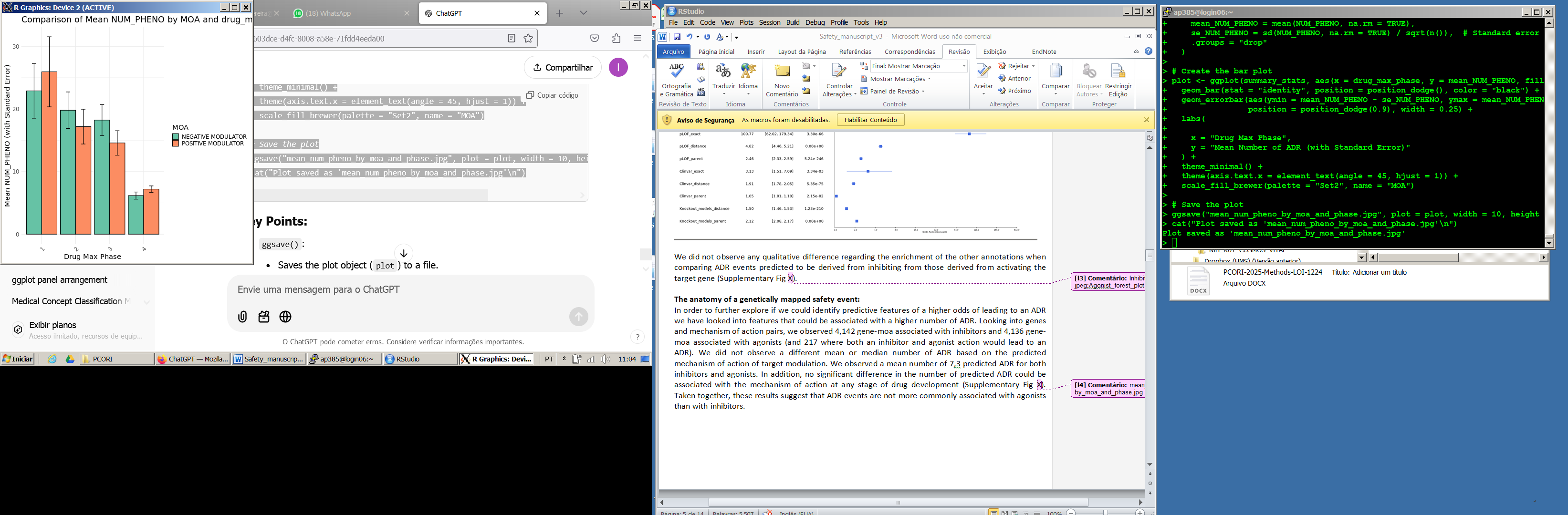


S2 Fig – Mean number of phenotypes by both mechanism of action and drug phase.

Supplement: S2 Fig — (DOCX) [file pgen.1012211.s002.docx]

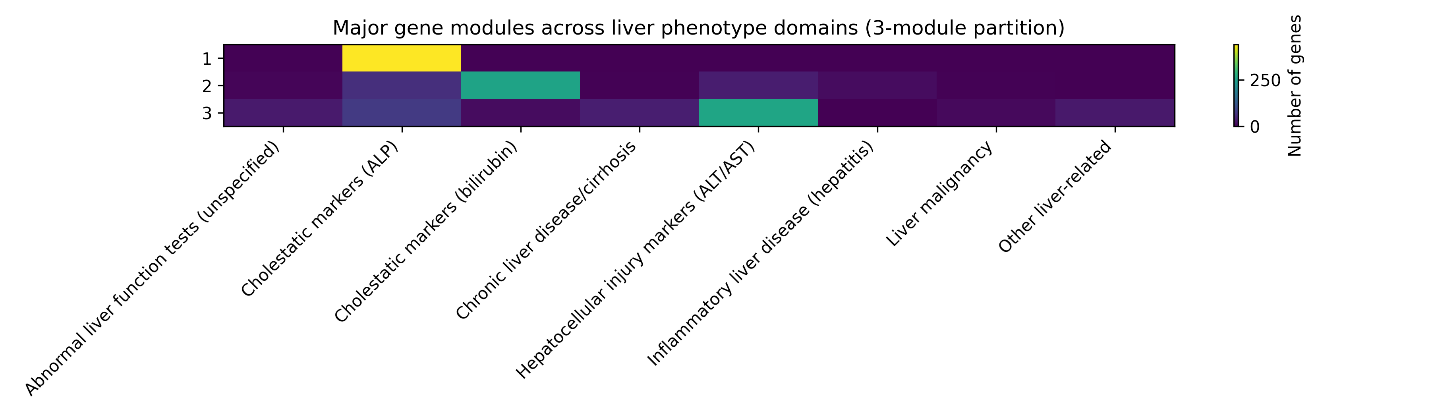


S3 Fig – Number of genes found in each community stratified by disease domains.

Supplement: S3 Fig — (DOCX) [file pgen.1012211.s003.docx]
