# Supplementary material for: A genomic-led strategy to anticipate drug safety effects
Source: PLoS Genet. 2026 Jul 16;22(7):e1012211. doi: 10.1371/journal.pgen.1012211 (PMC13375020; doi:10.1371/journal.pgen.1012211)
Supplement: S1 Text — (DOCX) [file pgen.1012211.s006.docx]

**Vignette Methods Section**

**Methods**

**Analysis Overview**

We implemented an integrative analytical framework to characterize liver-related gene modules, expand these modules using protein–protein interaction networks, map approved drugs to module genes, and evaluate associations with clinically reported hepatotoxicity. The workflow combines gene–phenotype association networks, network-based module expansion, drug–target mapping, and external hepatotoxicity annotations. All analyses were conducted using reproducible scripts, with inputs, intermediate files, and outputs archived in a structured analysis bundle.

**Liver phenotype selection and domain assignment**

To construct a liver-focused phenotype panel, we selected 18 liver-related traits from the full atlas based on direct relevance to clinical liver injury and routine biochemical assessment. Traits were manually grouped into seven liver-domain categories reflecting established clinical interpretation (e.g., hepatocellular injury, cholestatic injury, synthetic function). Domain assignment was performed prior to network analysis and was used solely to support interpretability of downstream results.

**Gene–Phenotype Network Construction and Module Detection**

Gene–phenotype associations were derived from the curated ADR atlas of genetic links to liver-related clinical biomarkers, including alanine aminotransferase (ALT), aspartate aminotransferase (AST), alkaline phosphatase (ALP), and bilirubin. These associations were represented as a bipartite network connecting genes to phenotypes. The network was projected onto the gene space, and community detection was performed using a modularity-based clustering algorithm to identify gene modules (Louvain). In the projected gene–gene network, edge weights corresponded to the number of shared liver phenotypes linking each gene pair. Three primary modules were identified, each dominated by a distinct liver injury domain corresponding to hepatocellular injury or cholestatic markers.

**Module-wise pathway enrichment analysis**

Pathway enrichment analysis was performed separately for each gene module using Enrichr. Reactome 2022, GO Biological Process 2023, and KEGG 2021 Human databases were queried. Pathways were considered significantly enriched at a false discovery rate (FDR) q ≤ 0.05.

**Pathway-level overlap across modules**

Pathway overlap across modules was assessed using two complementary approaches. First, exact pathway name matching was used to identify pathways significantly enriched in more than one module. Second, similarity between pathway labels was quantified using TF–IDF vectorization of pathway names followed by cosine similarity, with a similarity threshold of 0.75 used to define high-similarity pathway pairs.

**Module Expansion Using Protein–Protein Interaction Networks**

To evaluate the robustness and biological coverage of the initial modules, we expanded each module using protein–protein interaction data from the STRING database. Genes interacting with module members at a STRING combined confidence score ≥0.8 were added to the corresponding module. Expansion was performed independently for each module to preserve domain specificity. Pre- and post-expansion module characteristics were summarized, including module size, overlap between modules, and changes in intra- and inter-module connectivity.

**Drug–Target Mapping and Drug Severity Scoring**

Approved drugs were mapped to genes using curated drug–target annotations. For drugs targeting multiple genes, all gene–module mappings were retained. Drug-level severity scores were computed by aggregating module-level evidence across all mapped targets, resulting in a quantitative measure reflecting the extent and distribution of module involvement. Drugs were classified as single-module or multi-module depending on whether their targets mapped to one or multiple modules. Summary statistics describing drug mappings and severity score distributions were generated and stored as tabular outputs.

**LiverTox Annotation and Hepatotoxicity Assessment**

Mapped drugs were annotated using the LiverTox database to identify reported hepatotoxicity. LiverTox categories were harmonized into ordinal severity classes. Drugs sharing the same active compound were deduplicated, and the most severe LiverTox classification was retained for downstream analyses. Comparative analyses evaluated differences in hepatotoxicity prevalence between single-module and multi-module drugs, with sensitivity analyses restricted to higher-confidence LiverTox categories.

**Target-Profile Breadth and DILIrank 2.0 Stratification**

To quantify module engagement for each drug, we calculated a drug-level module entropy based on the distribution of mapped targets across liver injury modules. Entropy was computed using the normalized Shannon entropy of module membership probabilities, yielding a continuous measure of target-profile breadth. Drugs were stratified according to DILIrank 2.0 categories, and entropy distributions were compared descriptively across categories. For binary analyses, DILIrank categories were dichotomized into DILI-positive (Most-DILI-Concern or Less-DILI-Concern) versus DILI-negative (No-DILI-Concern), excluding Ambiguous-DILI-Concern drugs. These analyses were used solely to characterize differences in target dispersion patterns and were not used to redefine module assignments or severity scores.

**DILIrank 2.0 Resource Description**

DILIrank 2.0 was used as an external reference standard for classifying drugs by hepatotoxicity concern. Drugs were mapped to DILIrank 2.0 categories based on active compound names, with manual harmonization of synonyms where necessary. For drugs with multiple formulations or shared active ingredients, a single DILIrank 2.0 classification was assigned. DILIrank 2.0 categories were used for stratified analyses of drug-level properties, including module entropy, but were not used in module construction or expansion.

**Statistical analysis of drug-level hepatotoxicity associations**

Associations between module breadth and DILI status were evaluated using Fisher’s exact tests for contingency table analyses. Logistic regression models were fitted to estimate the association between DILI-positive status and number of modules targeted, adjusting for the total number of mapped target genes. Sensitivity analyses stratified drugs by total target gene count, including analyses restricted to drugs with exactly two mapped targets.
