# Supplementary material for: A genomic-led strategy to anticipate drug safety effects
Source: PLoS Genet. 2026 Jul 16;22(7):e1012211. doi: 10.1371/journal.pgen.1012211 (PMC13375020; doi:10.1371/journal.pgen.1012211)
Supplement: S2 Text — (DOCX) [file pgen.1012211.s007.docx]

**Severity classification of clinical phenotypes**

**Overview and objective**

We developed a deterministic, fully reproducible severity classification system to assign each clinical phenotype (including adverse event terms, diagnoses, laboratory or vital-sign phenotypes, and other phenotype descriptions) to an ordinal severity category intended to reflect likely clinical impact. The system was designed to (i) enforce a single, unique severity label for each unique phenotype string (i.e., the same phenotype text always receives the same severity category), (ii) depend on clinically interpretable, transparent rule sets rather than model-based inference, and (iii) enable external users to reproduce the classification exactly in their own phenotype lists. For each phenotype, the classifier returns both a categorical severity score on a four-level ordinal scale (1 = mild, 2 = moderate, 3 = severe, 4 = life-threatening) and a standardized, human-readable severity description aligned to the assigned category. This framework was applied consistently across (a) phenotype strings created using a “clinical phenotype” construct (for example, concatenating directionality such as High/Low with a phenotype description) and (b) free-text adverse event terms (for example, FAERS event names), using the same prioritized rule hierarchy to ensure comparable severity assignment across phenotype sources.

**Input preprocessing and phenotype normalization**

**Phenotype construction (when directionality is present)**

For datasets that include a direction-of-effect or safety signal indicator, we constructed a single “clinical phenotype” string by prefixing the phenotype description with a direction token such that Safety_signal = “up” is mapped to the prefix “High ” and Safety_signal = “down” is mapped to the prefix “Low ”; if Safety_signal is missing or not recognized, the phenotype description is retained without any prefix. Operationally, the resulting string is defined as clinical_phenotype = "<High/Low> <DESCRIPTION>", which preserves interpretability (e.g., “High creatinine” versus “Low creatinine”) while allowing the severity algorithm to operate on a single, standardized text field. To ensure deterministic rule matching, each phenotype string is normalized prior to evaluation by converting all characters to lowercase, trimming leading and trailing whitespace, and (recommended) collapsing repeated internal whitespace to a single space; missing or empty strings are treated as “unknown” and assigned the default severity category (2 = moderate).

**Ensuring one unique label per unique phenotype**

Severity was assigned at the level of the unique phenotype string rather than on a row-by-row basis: we first extracted the set of distinct phenotype strings, then applied the severity classifier to each unique string to generate severity_category and severity_description, and finally mapped these results back to the full dataset via exact string matching. This approach guarantees that every occurrence of the same phenotype text receives an identical severity label, thereby preventing within-phenotype inconsistencies that can arise when classifications are computed row-wise under context-dependent logic. Optionally, to improve portability across phenotype sources, users may additionally normalize common spelling variants (e.g., haemorrhage versus hemorrhage), although our implementation primarily relied on regular-expression pattern matching that already accommodates many such variants.

**Rule-based severity assignment**

**Severity definitions**

The four severity categories were defined as follows: category 1 (mild) corresponds to a benign condition, symptom, or biomarker/risk-factor measure that is typically self-limited or unlikely to require urgent medical care; category 2 (moderate) corresponds to a clinically meaningful condition or abnormality that may warrant evaluation and treatment but is not usually immediately life-threatening and does not generally imply major organ failure or irreversible harm; category 3 (severe) corresponds to major morbidity that is likely to require urgent care and/or hospitalization, confers substantial risk of irreversible harm (e.g., organ failure, major bleeding, malignancy), or represents a high-acuity diagnosis; and category 4 (life-threatening) corresponds to an immediate risk of death or rapid clinical deterioration requiring emergency intervention and/or intensive care.

**Rule hierarchy (priority order)**

We implemented a first-match-wins hierarchy in which each phenotype string is evaluated sequentially against rule sets ordered by clinical urgency; once a rule matches, the corresponding severity category is assigned and no subsequent rules are evaluated. The priority order was: life-threatening (4), severe (3), mild (1), structured laboratory/vital-sign subclasses (assigned as 1–3 depending on the analyte group), moderate diagnostic patterns (2), and finally fallback heuristics (assigned as 2 or 3 based on explicit severity cues, with a default of 2 when no cues are present). This ordering is deliberate because it prevents low-acuity tokens from overriding high-acuity diagnoses, ensuring, for example, that a phenotype containing “hemorrhage” is classified as at least severe regardless of any additional benign terms in the string.

**Clinical rule sets (regex-based)**

We then applied case-insensitive regular-expression matching to the normalized phenotype string, with the full implementation expressed as ordered lists of regex patterns. In brief, phenotypes were assigned to life-threatening severity (category 4) when they contained terms consistent with immediate mortality risk or ICU-level emergencies, including death/fatality (e.g., death, fatal, mortality), cardiorespiratory arrest (cardiac arrest, respiratory arrest, asystole), malignant ventricular arrhythmias (ventricular fibrillation, torsades, paroxysmal ventricular tachycardia), anaphylaxis or shock (anaphylaxis, anaphylactic shock, septic shock, shock), severe neurologic emergencies (coma, status epilepticus), catastrophic bleeding/CNS events (intracranial hemorrhage, subarachnoid hemorrhage), major vascular events (myocardial infarction, stroke, pulmonary embolism, aortic dissection), respiratory failure syndromes (respiratory failure, ARDS), severe cutaneous adverse reactions (toxic epidermal necrolysis/TEN, Stevens–Johnson), self-harm/overdose (suicide attempt, overdose), and acute metabolic emergencies (diabetic ketoacidosis/DKA, hyperosmolar states), reflecting the need for emergency stabilization. Phenotypes were assigned to severe severity (category 3) when they implied major morbidity, high hospitalization risk, irreversible harm, or other high-acuity diagnoses, including malignancy (malignancy/cancer/carcinoma, leukemia, lymphoma, myeloma), major organ failure or severe organ injury (heart failure, cardiomyopathy, severe valvular disease; renal failure, acute kidney injury, ESRD/dialysis; hepatic failure, fulminant hepatitis, hepatic necrosis; pancreatitis; and sepsis in the absence of shock), severe hematologic disorders (agranulocytosis, aplastic anemia, TTP, DIC), diagnosis-level cytopenias treated as clinical conditions rather than measurements (e.g., neutropenia, thrombocytopenia), severe bleeding or perforation syndromes (gastrointestinal hemorrhage/hemorrhage; intestinal/bowel/colon perforation), major thromboembolic events (DVT, thrombosis, with pulmonary embolism reserved for category 4), serious neuropsychiatric disorders (psychosis, delirium, and severe neurodegeneration such as dementia, Alzheimer’s disease, Parkinson’s disease, ALS), irreversible ocular harm (blindness, vision loss, retinal detachment, and glaucoma classified conservatively as severe given potential irreversible visual loss), and high-risk arrhythmias/dysrhythmias (arrhythmia, dysrhythmia, QT prolongation, with malignant ventricular rhythms classified as category 4), consistent with high-acuity clinical states and substantial long-term morbidity. Phenotypes were assigned to mild severity (category 1) when they aligned with benign symptoms, self-limited conditions, or low-acuity issues typically managed in the outpatient setting, including common symptoms (headache, nausea, vomiting, diarrhea, constipation, fatigue, dizziness), minor mucocutaneous findings (pruritus/itching, rhinitis, nasal congestion), sleep/sedation effects (insomnia, somnolence, drowsiness), musculoskeletal aches (myalgia, arthralgia), cosmetic or non-urgent conditions (alopecia, acne), minor ENT findings (cerumen, impacted cerumen), and peripheral edema when used in a symptom context without features suggesting cardiac decompensation. To reduce overassignment to “moderate” among quantitative phenotypes and clinical chemistry variables, we applied structured lab/vital subclasses (assigned as categories 1–3 depending on analyte group): electrolytes were assigned category 2 by default, with potassium abnormalities escalated to category 3 given arrhythmia risk; renal function markers (e.g., creatinine, eGFR, cystatin C) and liver injury/cholestasis markers (ALT/AST, alkaline phosphatase, bilirubin, GGT) were assigned category 2 as clinically meaningful indicators of potential organ injury; vital signs (blood pressure, heart rate) were assigned category 2; lipid and anthropometric measures (LDL/HDL/total cholesterol/triglycerides; BMI/weight/waist) were assigned category 1 because they typically represent risk-factor measures rather than acute harm in isolation; and generic measurement tokens (e.g., measurement/count/percentage) were assigned category 1 unless a more clinically meaningful analyte group matched. Within hematology, measurement-based abnormalities (e.g., “neutrophil count decreased”) were assigned category 2, whereas diagnosis-level cytopenias (e.g., “neutropenia”) were assigned category 3, reflecting differences in typical clinical management and acuity. Finally, if no higher-priority rule matched, phenotypes were assigned to moderate severity (category 2) when they contained patterns consistent with clinically meaningful but not typically immediately life-threatening diagnoses, including hypertension or hypotension as conditions, asthma, thyroid dysfunction, nephrolithiasis/urinary calculus, depression/anxiety, macular disease/cataract (vision impact but usually non-emergent), drug hypersensitivity and rash/urticaria excluding anaphylaxis, generic infections without sepsis/shock/pneumonia, and drug interaction terms treated as informational clinical safety events rather than physiologic collapse.

**Fallback heuristics and default assignment**

For phenotype strings that were not captured by the explicit rule sets, we applied conservative fallback heuristics to avoid systematic underestimation of clinically important events: if the normalized phenotype contained severity cue terms such as “failure,” “necrosis,” “metast,” “malign,” “recurrent,” “severe,” or “serious,” it was assigned category 3 (severe), whereas all remaining uncategorized phenotypes were assigned the default category 2 (moderate) to reflect potential clinical relevance in the absence of more specific information.

**Output fields and mapping**

For each unique phenotype string, we output two fields: a categorical severity label (severity_category ∈ {1,2,3,4}) and a standardized human-readable narrative (severity_description) derived from a fixed mapping to ensure consistency across datasets and analyses. The recommended standardized descriptions are: category 1, “Mild: benign condition or self-limited symptom; typically does not require urgent medical care.”; category 2, “Moderate: clinically meaningful condition or lab abnormality that may require evaluation and treatment.”; category 3, “Severe: major morbidity, likely to require urgent care/hospitalization, or risk of irreversible harm.”; and category 4, “Life-threatening: immediate risk of death or rapid deterioration; requires emergency intervention and/or intensive care.”
