# Supplementary material for: A genomic-led strategy to anticipate drug safety effects
Source: PLoS Genet. 2026 Jul 16;22(7):e1012211. doi: 10.1371/journal.pgen.1012211 (PMC13375020; doi:10.1371/journal.pgen.1012211)
Supplement: Attachment — Submitted filename: Reviewer comments.docx [file pgen.1012211.s010.docx]

Reviewer's Responses to Questions

**Comments to the Authors:
Please note here if the review is uploaded as an attachment.**

*Reviewer #1: I think this work represents a huge step forward in the field. It goes far beyond a simple academic paper; it's the outline of a navigation map for safer and more efficient drug development. The authors have built a solid bridge between large-scale human genetics and drug safety, providing an evidence-based framework to make the process more predictable and less risky. This is exactly the kind of "precision medicine" applied to drug discovery that we need.*

Thank you for your encouraging comments and interest in our work.

*Considerations

40% is good, but not 100%: The fact that 60% of predicted ADRs were not confirmed by FAERS requires explanation. The authors should provide food for thought for thought on the following:

1. False Positives: Intrinsic limitations of MR (pleiotropy).

2. Complex Pharmacology: The genetic model simulates lifelong inhibition/activation, not transient drug intake.*

Thank you for the comment. These are important points to consider and certainly, it is important to take these results into context. While they validate the approach, showing a significant enrichment of gene-trait pairs for previously described ADRs, it also brings into question whether the other 60% are potentially true, previously undescribed, ADRs or false-positive results.

Indeed, we have shown before that the positive predictive value of a gene-trait association identified using our approach does not necessarily have a positive predictive value that allows one to solely use this information to clearly predict an expected ADR from an approved drug. The information has to be used as an extra data point in a set of other available biological information from a particular gene or system to derive a decision process. Our aim with making the resource available is to add this extra point of, we believe, valuable information to the decision process.

We have added the suggested possibilities in the revised Discussion section (lines 374-382) and tried to highlight this important point as something the reader must consider when evaluating the provided evidence.

*3. FAERS is a notoriously noisy and incomplete database and may contain errors.*

Thank you for the comment. We describe the limitations of FAERS in the Limitations and Future Directions section of the discussion, specifically in the second paragraph. We have also tried to use FAERS datasets that have been used by previous studies so that there is consistency in the dataset being used (please see revised Methods section). Finally, in our newly added liver toxicity vignette we have also used two other curated data resources for ADR: LiverTox and DILIrank 2.0.

*4. How well is the method predicting rare but serious adverse events (e.g., idiosyncratic hepatotoxicity)? Statistical power may be a limitation here.*

We agree that this is an important point. We have added language in the revised Discussion section on the problem of detecting rare, idiosyncratic, ADRs using the current framework. In this scenario, not only statistical power, but also the biological causation model (rare genetic variants with large effect sizes) is not covered in our approach.

*5. How to translate a "genetic safety signal" into a binary "proceed/do not proceed" decision? Some signals may be acceptable for severe diseases, but not for mild ones. A risk-benefit framework is needed.*

Thank you for the comment. Similar to the severity annotation that we have added to the FAERS events, we have also added extra columns to the safety atlas which can be found in supplementary table 10. This is also referenced in the manuscript at lines 410-412. Our intent is to allow the reader to also investigate potential severity associated with the detected gene-trait associations. We acknowledge that this does not completely provide a risk-benefit framework for drug development and is instead intended to inform early-stage target prioritization and hypothesis generation rather than serve as a prescriptive decision rule. We agree that specific analysis for each different clinical scenario is warranted. We have also added this important point in the revised Discussion section.

*6. How can this atlas be made available to the scientific community and industry? An interactive web portal would be an incredibly powerful tool.*

We completely agree. After this suggestion, we have developed an interactive web visualization tool that provides filtering by phenotype, gene, drug phase, and drug mechanism of action. This resource also provides the ability to download a table of the user selected results. A version of this tool can be found here: <https://shiny.parse-health.org/safety/>. We will continue to develop better functionalities into the tool in the future and would like to make the tool available to the community linked to the current manuscript.

*7. The system does not account for non-translatable RNAs, which could significantly impact drug response.*

This is an important point. The reviewer is correct and we have only addressed protein-coding genes. This is a limitation not only of our study but on the use of comprehensive Mendelian Randomization analysis for drug-phenotype prediction. This is even more evident with the current trend of prioritizing pQTLs as opposed to eQTLs. We have added this important point in the revised text at lines 478-480.

*Reviewer #2: It looks like the references in the main text of the manuscript do not correspond to the list of references at the end of the manuscript. For example, in the text, eQTLGen is cited as ref 25, but the correct one should be ref 9. After a brief check, it looks like most (if not all) of the reference numbers in the text are wrong. Unfortunately I will not be able to review the manuscript in detail before the references have been fixed, as it is currently impossible to check if the relevant literature has been cited in appropriate places.*

We deeply apologize for this mistake. We have revised all references and hope they are correctly rendered in the revised version of the manuscript.

*Reviewer #3: This is a very ambitious and timely study that proposes a genome-wide Mendelian randomisation (MR) approach to predict adverse drug reactions (ADRs) using genetic instruments across large datasets like the Million Veteran Program, UK Biobank and FinnGen. The authors integrate genetic proxies for gene expression and protein levels with drug mechanisms of action, creating what they call a safety “atlas” of predicted ADRs. I found the scope of the work impressive and the idea of combining MR with pharmacological annotation is quite innovative. It’s encouraging that the authors were able to validate some of their predicted gene-ADR links using FAERS data as well as clinical trials that were terminated for safety reasons, which strengthens the credibility of their method.*

Thank you very much for your interest in our work.

*That said, there are several areas that I feel need more attention before this manuscript is ready for publication.*

*First, the “safety signal metrics” mentioned in the Results are not defined; while they may be familiar to some readers, it is unclear how these signals were quantified or prioritised, which could make interpretation difficult for many.*

Thank you for your comment. This is an important point. Previous work in the area have mainly used gene-trait associations as a proxy for detecting potential safety concerns. Here, we have also taken advantage of the directionality of effect provided by Mendelian Randomization and a particular directionality considered to warrant clinical attention. For this, we have categorized each study trait in regards to what direction the odds associated with the trait should go as for considering the association an “unwanted” association, and, hence, potentially concerning. This classification was curated by two physicians (ACP and JMG) and described in Supplementary Table 1.

We have added new language in the revised Methods section at lines 627-647 to make this step of our analysis clear to the reader and we make the entire matrix fully available to readers so they can reproduce the procedure.

*Also, one of the p-value thresholds is shown as “XXX” in the Materials and Methods (under eQTLGen subsection), which looks like a placeholder and should be fixed and I think you should write a specific p-value.*

We apologize. This has been fixed in the revised manuscript.

*More broadly, I’m concerned that the authors did not perform any colocalisation analysis (or fine-mapping analyses?) to confirm that the genetic variant associated with expression or protein level is also driving the clinical trait association (i.e., the same causal variant drives both the QTL and the trait association). Without this,the causal link can be questionable due to linkage or horizontal pleiotropy (i.e., through through nearby genes); although cis-QTL instruments reduce pleiotropy, cis variants can still influence multiple genes. Even if such analyses are not feasible at scale, I think the authors should acknowledge this limitation clearly.*

We thank the reviewer for their comment. We have conducted extensive colocalization analysis between all significant gene-trait pairs. These results are available in Supplementary Table 2. We also added these new results in the revised Results section. Importantly, as the reviewer has pointed, several factors can explain a significant MR results with concomitant negative colocalization (in fact several of the confirmatory gene-trait associations that are also described in FAERS and other databases failed to show colocalization in our data), and, as a result, we did not discard gene-trait pairs with no evidence of colocalization, but left this at the reader’s discretion pointing this as an important intermediary step that can be used to move forward specific gene-traits for downstream study. This important point has been added in the revised Discussion section at lines 649-657.

*Also, some results are presented in summary (e.g. “1,093 that are predicted to be associated with an ADR for an approved drug.”) and I think that some details on effect sizes or the range of odds ratios can be useful.*

Thank you for your suggestion. We have added some descriptive data on the results section, but also provide individual gene-trait effect sizes and errors in Supplementary Table 1.

*In terms of phenotype mapping, manually clustering traits like “platelet count decreased” and “thrombocytopenia” makes sense, but it introduces subjectivity and it would be stronger if the authors used standard vocabularies like MedDRA.*

Thank you for your suggestion. We have discussed this point extensively in our group and we acknowledge that medical concept ontologies also don’t fully capture the relationships and correlation between phenotypes. The reviewer is correct that the clustering scheme is subjective and readers might decide to use their own clusterization. We make this possible by providing all gene-trait associations. Clusterization was just used to create the enrichment statistics to showcase the approach and provide evidence that it is able to capture relevant clinical information.

*I also felt the manuscript would benefit a lot from walking through one or two example gene-MoA-ADR predictions in the main text, showing how the MR evidence maps to a known drug effect. The resource is large, but I believe this can help grasp what a typical case looks like in practice.*

Thank you for this suggestion. We have added an extensive new section of the manuscript where we describe one of the many uses the dataset can be used to deliver new hypothesis and derive new clinical tools. In this new section, we provide a detailed walk-thru the mapping of liver toxicity using a series of related clinical phenotypes. We show how we can derive different axis of toxicity and how this information is able to predicts liver toxicity in approved drugs. It also suggests that several closely related genes might also be associated with liver toxicity and that new drugs targeting these genes might pose an increased risk of this outcome.

*Another point worth mentioning is that only ~40% of the predicted ADRs for approved drugs were found in FAERS, which is still a decent result - but it would help to discuss what might explain the remaining 60%. Are these possibly novel ADRs? or been false positives? A short discussion with examples would add balance. Also, although FAERS data was filtered, it has known limitations (like underreporting), so I suggest mentioning those more explicitly in the Discussion section.*

Thank you. These points were also raised by Reviewer#1 (please see response above). We have added some discussion acknowledging the potential limitations and on how these should be approached in future research.

*The analysis is limited to European ancestry populations, which is understandable for LD reasons or meet the LD assumptions, but this should also be discussed more openly as a limitation, since ADR risks can differ across populations.*

We agree with this point. It is important to convey the idea that our analysis should not be used as a proxy of ADR incidence in different ancestry groups. It should be used as a way to flag, and anticipate, potential ADR during the drug development process. This is not to say that conducting the same approach in different ancestry might also unveil a number of other significant gene-trait pairs that should also be used to predict ADRs for future drugs. We have added this nuanced point in the revised Discussion section at lines 499-511.

*Lastly, the way the authors infer mechanism of action (positive vs. negative modulation) from MR beta signs may need to be explained more clearly. It’s a nice idea, but readers may not all follow the logic of mapping a genetic increase in expression to an activator drug, for example.*

We have added a new subsection called “Inferring Direction of Pharmacological Modulation from Mendelian Randomization” in the Methods section explaining in detail the process and the assumptions of this approach.

*Overall, I think this is a highly promising study and the authors are addressing a really important area in pharmacogenomics. The concept is solid and the dataset is rich, but a few key methodological clarifications and examples would make the work stronger and more accessible. I’m recommending a major revision, and I hope the authors will take the chance to improve and clarify their analysis. I’d be happy to see a revised version once these points are addressed.*

Thank you very much. We appreciate your careful consideration and thoughtful comments. We believe the extensive modifications in the revised manuscript have significantly improved the work and we hope it is interesting to Plos Genetics readers.
