# Supplementary material for: A genomic-led strategy to anticipate drug safety effects
Source: PLoS Genet. 2026 Jul 16;22(7):e1012211. doi: 10.1371/journal.pgen.1012211 (PMC13375020; doi:10.1371/journal.pgen.1012211)
Supplement: Attachment — Submitted filename: Reviewer_comments_R2.docx [file pgen.1012211.s011.docx]

*First, I can see the Supporting Information legends in the revised file, but the supplementary tables themselves do not appear to be included in the PDF I received. Could the authors/journal please provide the supplementary tables (and any associated files) so I can verify the definitions and contents referenced in the text (e.g., the safety-signal metrics tables and colocalisation outputs)?*

The supplementary tables were most likely not included in the pdf as they are aggregated in an excel workbook. The journal will most likely need to provide the supplementary tables.

*Second, is it possible make the definition of the “safety signal metrics” and the curated “unwanted direction” mapping explicit and easy to find in the main Methods (not only by pointing to Supplementary Tables)? including a brief note that this is curated and therefore partly subjective even if reproducible can be helpful.*

Thank you for this comment. We have added a paragraph in the Methods section explicitly defining the safety signal as a function of unwanted direction. We also added language highlighting the subjective nature of our definition. This can be found on lines 658-667 of the revised manuscript with track changes on.

*Third, please ensure the final submission is a fully clean version and carefully check for residual marked-up/typographical artefacts (for example, an incorrect “258%” appears in the highlighted revision file).*

We thank the reviewer for this comment. In the revised version of the manuscript with track changes on, we see the 8 that is referenced has been crossed out indicating that it has been deleted. In the version of the manuscript with all changes accepted it reads “25%”.
