# Supplementary material for: A genomic-led strategy to anticipate drug safety effects
Source: PLoS Genet. 2026 Jul 16;22(7):e1012211. doi: 10.1371/journal.pgen.1012211 (PMC13375020; doi:10.1371/journal.pgen.1012211)
Supplement: Peer review history

# Peer review: Decision Letter 0

**DOI:** 10.1371/journal.pgen.1012211.r001

**Authors:** Gregory Cooper, Stuart Scott

9 Nov 2025

PGENETICS-D-25-01087

A genomic-led strategy to anticipate drug safety effects

PLOS Genetics

Dear Dr. Ferolito,

Thank you for submitting your manuscript to PLOS Genetics. After careful consideration, we feel that it has merit but does not fully meet PLOS Genetics's publication criteria as it currently stands. Therefore, we invite you to submit a revised version of the manuscript that addresses the points raised during the review process.

Please submit your revised manuscript by Jan 08 2026 11:59PM. If you will need more time than this to complete your revisions, please reply to this message or contact the journal office at plosgenetics@plos.org. When you're ready to submit your revision, log on to https://www.editorialmanager.com/pgenetics/ and select the 'Submissions Needing Revision' folder to locate your manuscript file.

Please include the following items when submitting your revised manuscript:

* A rebuttal letter that responds to each point raised by the editor and reviewer(s). You should upload this letter as a separate file labeled 'Response to Reviewers'. This file does not need to include responses to any formatting updates and technical items listed in the 'Journal Requirements' section below.

* A marked-up copy of your manuscript that highlights changes made to the original version. You should upload this as a separate file labeled 'Revised Manuscript with Track Changes'.

* An unmarked version of your revised paper without tracked changes. You should upload this as a separate file labeled 'Manuscript'.

If you would like to make changes to your financial disclosure, competing interests statement, or data availability statement, please make these updates within the submission form at the time of resubmission. Guidelines for resubmitting your figure files are available below the reviewer comments at the end of this letter.

We look forward to receiving your revised manuscript.

Kind regards,

Stuart A Scott, PhD

Academic Editor

PLOS Genetics

Gregory Cooper

Section Editor

PLOS Genetics

Aimée Dudley

Editor-in-Chief

PLOS Genetics

Anne Goriely

Editor-in-Chief

PLOS Genetics

**Additional Editor Comments:**

Thank you for your manuscript submission, which the Reviewers and Editors have reviewed and are providing comments to address with a revised version.

**Journal Requirements:**

If the reviewer comments include a recommendation to cite specific previously published works, please review and evaluate these publications to determine whether they are relevant and should be cited. There is no requirement to cite these works unless the editor has indicated otherwise.

1) Please ensure that the CRediT author contributions listed for every co-author are completed accurately and in full.

At this stage, the following Authors/Authors require contributions: Brian Richard Ferolito, Andrea RVR Horimoto, Kai Gravel-Pucillo, Daniel J Golden, Hesam Dashti, Claudia Giambartolomei, Danielle Rasooly, Rachael Matty, Liam Gaziano, Yakov Tsepilov, Lauren Costa, Nicole Kosik, Harris Ioannidis, Mohd Karim, Giovanna Winicki, Fiona Hunter, Claudia Langenberg, John C Whittaker, Million Veteran Program, Tianxi Cai, Gina M Peloso, Barbara Zdrazil, Maya Ghoussaini, Andrew R Leach, Sumitra Muralidhar, Ines A Smit, Juan P Casas, J Michael Gaziano, Kelly Cho, and Alexandre C Pereira. Please ensure that the full contributions of each author are acknowledged in the "Add/Edit/Remove Authors" section of our submission form.

The list of CRediT author contributions may be found here: https://journals.plos.org/plosgenetics/s/authorship#loc-author-contributions

2) Please provide an Author Summary. This should appear in your manuscript between the Abstract (if applicable) and the Introduction, and should be 150-200 words long. The aim should be to make your findings accessible to a wide audience that includes both scientists and non-scientists. Sample summaries can be found on our website under Submission Guidelines:

https://journals.plos.org/plosgenetics/s/submission-guidelines#loc-parts-of-a-submission

3) Please upload all main figures as separate Figure files in .tif or .eps format. For more information about how to convert and format your figure files please see our guidelines:

https://journals.plos.org/plosgenetics/s/figures

4) We have noticed that you have uploaded Supporting Information files, but you have not included a list of legends. Please add a full list of legends for your Supporting Information files after the references list.

5) Please provide a detailed Financial Disclosure statement. This is published with the article. It must therefore be completed in full sentences and contain the exact wording you wish to be published.

1) Please clarify all sources of financial support for your study. List the grants, grant numbers, and organizations that funded your study, including funding received from your institution. Please note that suppliers of material support, including research materials, should be recognized in the Acknowledgements section rather than in the Financial Disclosure

2) State the initials, alongside each funding source, of each author to receive each grant. For example: "This work was supported by the National Institutes of Health (####### to AM; ###### to CJ) and the National Science Foundation (###### to AM)."

3) State what role the funders took in the study. If the funders had no role in your study, please state: "The funders had no role in study design, data collection and analysis, decision to publish, or preparation of the manuscript."

4) If any authors received a salary from any of your funders, please state which authors and which funders..

If you did not receive any funding for this study, please simply state: u201cThe authors received no specific funding for this work.u201d

6) Your current Financial Disclosure states, "The author(s) received no specific funding for this work.".

However, your funding information on the submission form indicates recieving fund from U.S. Department of Veterans Affairs.

Please indicate by return email the full and correct funding information for your study and confirm the order in which funding contributions should appear. Please be sure to indicate whether the funders played any role in the study design, data collection and analysis, decision to publish, or preparation of the manuscript.

7) Please send a completed 'Competing Interests' statement, including any COIs declared by your co-authors. If you have no competing interests to declare, please state "The authors have declared that no competing interests exist". Otherwise please declare all competing interests beginning with the statement "I have read the journal's policy and the authors of this manuscript have the following competing interests"

**Reviewers' comments:**

Reviewer's Responses to Questions

**Comments to the Authors:**

**Please note here if the review is uploaded as an attachment.**

Reviewer #1: I think this work represents a huge step forward in the field. It goes far beyond a simple academic paper; it's the outline of a navigation map for safer and more efficient drug development. The authors have built a solid bridge between large-scale human genetics and drug safety, providing an evidence-based framework to make the process more predictable and less risky. This is exactly the kind of "precision medicine" applied to drug discovery that we need.

Considerations

40% is good, but not 100%: The fact that 60% of predicted ADRs were not confirmed by FAERS requires explanation. The authors should provide food for thought for thought on the following:

1. False Positives: Intrinsic limitations of MR (pleiotropy).

2. Complex Pharmacology: The genetic model simulates lifelong inhibition/activation, not transient drug intake.

3. FAERS is a notoriously noisy and incomplete database and may contain errors.

4. How well is the method predicting rare but serious adverse events (e.g., idiosyncratic hepatotoxicity)? Statistical power may be a limitation here.

5. How to translate a "genetic safety signal" into a binary "proceed/do not proceed" decision? Some signals may be acceptable for severe diseases, but not for mild ones. A risk-benefit framework is needed.

6. How can this atlas be made available to the scientific community and industry? An interactive web portal would be an incredibly powerful tool.

7. The system does not account for non-translatable RNAs, which could significantly impact drug response.

Reviewer #2: It looks like the references in the main text of the manuscript do not correspond to the list of references at the end of the manuscript. For example, in the text, eQTLGen is cited as ref 25, but the correct one should be ref 9. After a brief check, it looks like most (if not all) of the reference numbers in the text are wrong. Unfortunately I will not be able to review the manuscript in detail before the references have been fixed, as it is currently impossible to check if the relevant literature has been cited in appropriate places.

Reviewer #3: This is a very ambitious and timely study that proposes a genome-wide Mendelian randomisation (MR) approach to predict adverse drug reactions (ADRs) using genetic instruments across large datasets like the Million Veteran Program, UK Biobank and FinnGen. The authors integrate genetic proxies for gene expression and protein levels with drug mechanisms of action, creating what they call a safety “atlas” of predicted ADRs. I found the scope of the work impressive and the idea of combining MR with pharmacological annotation is quite innovative. It’s encouraging that the authors were able to validate some of their predicted gene-ADR links using FAERS data as well as clinical trials that were terminated for safety reasons, which strengthens the credibility of their method.

That said, there are several areas that I feel need more attention before this manuscript is ready for publication. First, the “safety signal metrics” mentioned in the Results are not defined; while they may be familiar to some readers, it is unclear how these signals were quantified or prioritised, which could make interpretation difficult for many. Also,one of the p-value thresholds is shown as “XXX” in the Materials and Methods (under eQTLGen subsection), which looks like a placeholder and should be fixed and I think you should write a specific p-value. More broadly, I’m concerned that the authors did not perform any colocalisation analysis (or fine-mapping analyses?) to confirm that the genetic variant associated with expression or protein level is also driving the clinical trait association (i.e., the same causal variant drives both the QTL and the trait association). Without this,the causal link can be questionable due to linkage or horizontal pleiotropy (i.e., through through nearby genes); although cis-QTL instruments reduce pleiotropy, cis variants can still influence multiple genes. Even if such analyses are not feasible at scale, I think the authors should acknowledge this limitation clearly.

Also, some results are presented in summary (e.g. “1,093 that are predicted to be associated with an ADR for an approved drug.”) and I think that some details on effect sizes or the range of odds ratios can be useful.

In terms of phenotype mapping, manually clustering traits like “platelet count decreased” and “thrombocytopenia” makes sense, but it introduces subjectivity and it would be stronger if the authors used standard vocabularies like MedDRA. I also felt the manuscript would benefit a lot from walking through one or two example gene-MoA-ADR predictions in the main text, showing how the MR evidence maps to a known drug effect. The resource is large, but I believe this can help grasp what a typical case looks like in practice.

Another point worth mentioning is that only ~40% of the predicted ADRs for approved drugs were found in FAERS, which is still a decent result - but it would help to discuss what might explain the remaining 60%. Are these possibly novel ADRs? or been false positives? A short discussion with examples would add balance. Also, although FAERS data was filtered, it has known limitations (like underreporting), so I suggest mentioning those more explicitly in the Discussion section.

The analysis is limited to European ancestry populations, which is understandable for LD reasons or meet the LD assumptions, but this should also be discussed more openly as a limitation, since ADR risks can differ across populations. Lastly, the way the authors infer mechanism of action (positive vs. negative modulation) from MR beta signs may need to be explained more clearly. It’s a nice idea, but readers may not all follow the logic of mapping a genetic increase in expression to an activator drug, for example.

Overall, I think this is a highly promising study and the authors are addressing a really important area in pharmacogenomics. The concept is solid and the dataset is rich, but a few key methodological clarifications and examples would make the work stronger and more accessible. I’m recommending a major revision, and I hope the authors will take the chance to improve and clarify their analysis. I’d be happy to see a revised version once these points are addressed.

**********

**Have all data underlying the figures and results presented in the manuscript been provided?**

Large-scale datasets should be made available via a public repository as described in the *PLOS Genetics*  data availability policy, and numerical data that underlies graphs or summary statistics should be provided in spreadsheet form as supporting information.

Reviewer #1: Yes

Reviewer #2: None

Reviewer #3: **No:** Partially:

The authors state that they plan to share their Mendelian Randomisation results in Dryad upon publication, and they have made interim data available via Dropbox for peer review. However, at present, the main atlas and related summary statistics are not yet publicly deposited in a permanent repository. This should be addressed before publication to meet PLOS Genetics’ data sharing requirements.

**********

PLOS authors have the option to publish the peer review history of their article (what does this mean?). If published, this will include your full peer review and any attached files.

If you choose “no”, your identity will remain anonymous but your review may still be made public.

**Do you want your identity to be public for this peer review?**  For information about this choice, including consent withdrawal, please see our Privacy Policy.

Reviewer #1: **Yes:** Giuseppe Novelli

Reviewer #2: No

Reviewer #3: **Yes:** Kinan Mokbel

**Figure resubmission:**

While revising your submission, we strongly recommend that you use PLOS’s NAAS tool (https://ngplosjournals.pagemajik.ai/artanalysis) to test your figure files. NAAS can convert your figure files to the TIFF file type and meet basic requirements (such as print size, resolution), or provide you with a report on issues that do not meet our requirements and that NAAS cannot fix.

After uploading your figures to PLOS’s NAAS tool - https://ngplosjournals.pagemajik.ai/artanalysis, NAAS will process the files provided and display the results in the "Uploaded Files" section of the page as the processing is complete. If the uploaded figures meet our requirements (or NAAS is able to fix the files to meet our requirements), the figure will be marked as "fixed" above. If NAAS is unable to fix the files, a red "failed" label will appear above. When NAAS has confirmed that the figure files meet our requirements, please download the file via the download option, and include these NAAS processed figure files when submitting your revised manuscript.

**Reproducibility:**

To enhance the reproducibility of your results, we recommend that authors of applicable studies deposit laboratory protocols in protocols.io, where a protocol can be assigned its own identifier (DOI) such that it can be cited independently in the future. Additionally, PLOS ONE offers an option to publish peer-reviewed clinical study protocols. Read more information on sharing protocols at https://plos.org/protocols?utm_medium=editorial-email&utm_source=authorletters&utm_campaign=protocols

# Author comment: Author response to Decision Letter 1

**DOI:** 10.1371/journal.pgen.1012211.r002

11 Feb 2026

Attachment Submitted filename: Reviewer comments.docx

# Peer review: Decision Letter 1

**DOI:** 10.1371/journal.pgen.1012211.r003

**Authors:** Gregory Cooper, Stuart Scott, Gregory Cooper, Stuart Scott

19 Mar 2026

PGENETICS-D-25-01087R1

A genomic-led strategy to anticipate drug safety effects

PLOS Genetics

Dear Dr. Pereira,

Thank you for submitting your manuscript to PLOS Genetics. After careful consideration, we feel that it has merit but does not fully meet PLOS Genetics's publication criteria as it currently stands. Therefore, we invite you to submit a revised version of the manuscript that addresses the points raised during the review process.

Please submit your revised manuscript within by Apr 18 2026 11:59PM. If you will need more time than this to complete your revisions, please reply to this message or contact the journal office at plosgenetics@plos.org. When you're ready to submit your revision, log on to https://www.editorialmanager.com/pgenetics/ and select the 'Submissions Needing Revision' folder to locate your manuscript file.

Please include the following items when submitting your revised manuscript:

* A letter that responds to each point raised by the editor and reviewer(s). You should upload this letter as a separate file labeled 'Response to Reviewers'. This file does not need to include responses to formatting updates and technical items listed in the 'Journal Requirements' section below.

* A marked-up copy of your manuscript that highlights changes made to the original version. You should upload this as a separate file labeled 'Revised Manuscript with Track Changes'.

* An unmarked version of your revised paper without tracked changes. You should upload this as a separate file labeled 'Manuscript'.

If you would like to make changes to your financial disclosure, competing interests statement, or data availability statement, please make these updates within the submission form at the time of resubmission. Guidelines for resubmitting your figure files are available below the reviewer comments at the end of this letter.

We look forward to receiving your revised manuscript.

Kind regards,

Stuart A Scott, PhD

Academic Editor

PLOS Genetics

Gregory Cooper

Section Editor

PLOS Genetics

Aimée Dudley

Editor-in-Chief

PLOS Genetics

Anne Goriely

Editor-in-Chief

PLOS Genetics

**Reviewers' comments:**

Reviewer's Responses to Questions

**Comments to the Authors:**

**Please note here if the review is uploaded as an attachment.**

Reviewer #1: The authors have substantially modified the manuscript in accordance with the reviewers' suggestions and therefore I now consider it acceptable.

Reviewer #3: The authors have responded carefully to my prior comments and the revision substantially strengthens the manuscript. I have only minor remaining requests before acceptance:

First, I can see the Supporting Information legends in the revised file, but the supplementary tables themselves do not appear to be included in the PDF I received. Could the authors/journal please provide the supplementary tables (and any associated files) so I can verify the definitions and contents referenced in the text (e.g., the safety-signal metrics tables and colocalisation outputs)?

Second, is it possible make the definition of the “safety signal metrics” and the curated “unwanted direction” mapping explicit and easy to find in the main Methods (not only by pointing to Supplementary Tables)? including a brief note that this is curated and therefore partly subjective even if reproducible can be helpful.

Third, please ensure the final submission is a fully clean version and carefully check for residual marked-up/typographical artefacts (for example, an incorrect “258%” appears in the highlighted revision file).

Subject to these minor points, I support publication.

**********

**Have all data underlying the figures and results presented in the manuscript been provided?**

Large-scale datasets should be made available via a public repository as described in the *PLOS Genetics*  data availability policy, and numerical data that underlies graphs or summary statistics should be provided in spreadsheet form as supporting information.

Reviewer #1: None

Reviewer #3: **No:**

**********

PLOS authors have the option to publish the peer review history of their article (what does this mean?). If published, this will include your full peer review and any attached files.

If you choose “no”, your identity will remain anonymous but your review may still be made public.

**Do you want your identity to be public for this peer review?**  For information about this choice, including consent withdrawal, please see our Privacy Policy.

Reviewer #1: **Yes:** Giuseppe Novelli

Reviewer #3: **Yes:** Kinan Mokbel

[NOTE: If reviewer comments were submitted as an attachment file, they will be attached to this email and accessible via the submission site. Please log into your account, locate the manuscript record, and check for the action link "View Attachments". If this link does not appear, there are no attachment files.]

**Figure resubmission:**

-->While revising your submission, we strongly recommend that you use PLOS’s NAAS tool (https://ngplosjournals.pagemajik.ai/artanalysis) to test your figure files. NAAS can convert your figure files to the TIFF file type and meet basic requirements (such as print size, resolution), or provide you with a report on issues that do not meet our requirements and that NAAS cannot fix.

After uploading your figures to PLOS’s NAAS tool - https://ngplosjournals.pagemajik.ai/artanalysis, NAAS will process the files provided and display the results in the "Uploaded Files" section of the page as the processing is complete. If the uploaded figures meet our requirements (or NAAS is able to fix the files to meet our requirements), the figure will be marked as "fixed" above. If NAAS is unable to fix the files, a red "failed" label will appear above. When NAAS has confirmed that the figure files meet our requirements, please download the file via the download option, and include these NAAS processed figure files when submitting your revised manuscript.

**Reproducibility:**

To enhance the reproducibility of your results, we recommend that authors deposit laboratory protocols in protocols.io, where a protocol can be assigned its own identifier (DOI) such that it can be cited independently in the future. Additionally, PLOS ONE offers an option to publish peer-reviewed clinical study protocols. Read more information on sharing protocols at https://plos.org/protocols?utm_medium=editorial-email&utm_source=authorletters&utm_campaign=protocols

# Author comment: Author response to Decision Letter 2

**DOI:** 10.1371/journal.pgen.1012211.r004

29 May 2026

Attachment Submitted filename: Reviewer_comments_R2.docx

# Editor report: Decision Letter 2

**DOI:** 10.1371/journal.pgen.1012211.r005

**Authors:** Gregory Cooper, Stuart Scott, Gregory Cooper, Stuart Scott, Gregory Cooper, Stuart Scott

8 Jun 2026

Dear Dr Pereira,

We are pleased to inform you that your manuscript entitled "A genomic-led strategy to anticipate drug safety effects" has been editorially accepted for publication in PLOS Genetics. Congratulations!

Before your submission can be formally accepted and sent to production you will need to complete our formatting changes, which you will receive in a follow up email. Please be aware that it may take several days for you to receive this email; during this time no action is required by you. Please note: the accept date on your published article will reflect the date of this provisional acceptance, but your manuscript will not be scheduled for publication until the required changes have been made.

Once your paper is formally accepted, an uncorrected proof of your manuscript will be published online ahead of the final version, unless you’ve already opted out via the online submission form. If, for any reason, you do not want an earlier version of your manuscript published online or are unsure if you have already indicated as such, please let the journal staff know immediately at plosgenetics@plos.org.

In the meantime, please log into Editorial Manager at https://www.editorialmanager.com/pgenetics/, click the "Update My Information" link at the top of the page, and update your user information to ensure an efficient production and billing process. Note that PLOS requires an ORCID iD for all corresponding authors. Therefore, please ensure that you have an ORCID iD and that it is validated in Editorial Manager. To do this, go to ‘Update my Information’ (in the upper left-hand corner of the main menu), and click on the Fetch/Validate link next to the ORCID field.  This will take you to the ORCID site and allow you to create a new iD or authenticate a pre-existing iD in Editorial Manager.

If you have a press-related query, or would like to know about making your underlying data available (as you will be aware, this is required for publication), please see the end of this email. If your institution or institutions have a press office, please notify them about your upcoming article at this point, to enable them to help maximise its impact. Inform journal staff as soon as possible if you are preparing a press release for your article and need a publication date.

Thank you again for supporting open-access publishing; we are looking forward to publishing your work in PLOS Genetics!

Yours sincerely,

Stuart A Scott, PhD

Academic Editor

PLOS Genetics

Gregory Cooper

Section Editor

PLOS Genetics

Aimée Dudley

Editor-in-Chief

PLOS Genetics

Anne Goriely

Editor-in-Chief

PLOS Genetics

www.plosgenetics.org

BlueSky: @plos.bsky.social

----------------------------------------------------

Comments from the reviewers (if applicable):

Thank you again for revising your manuscript in accord with the additional review comments, and congratulations on your work.

- Editors

----------------------------------------------------

**Data Deposition**

If you have submitted a Research Article or Front Matter that has associated data that are not suitable for deposition in a subject-specific public repository (such as GenBank or ArrayExpress), one way to make that data available is to deposit it in the Dryad Digital Repository. As you may recall, we ask all authors to agree to make data available; this is one way to achieve that. A full list of recommended repositories can be found on our website.

The following link will take you to the Dryad record for your article, so you won't have to re‐enter its bibliographic information, and can upload your files directly:

http://datadryad.org/submit?journalID=pgenetics&manu=PGENETICS-D-25-01087R2

More information about depositing data in Dryad is available at http://www.datadryad.org/depositing. If you experience any difficulties in submitting your data, please contact help@datadryad.org for support.

Additionally, please be aware that our data availability policy requires that all numerical data underlying display items are included with the submission, and you will need to provide this before we can formally accept your manuscript, if not already present.

----------------------------------------------------

**Press Queries**

If you or your institution will be preparing press materials for this manuscript, or if you need to know your paper's publication date for media purposes, please inform the journal staff as soon as possible so that your submission can be scheduled accordingly. Your manuscript will remain under a strict press embargo until the publication date and time. This means an early version of your manuscript will not be published ahead of your final version. PLOS Genetics may also choose to issue a press release for your article. If there's anything the journal should know or you'd like more information, please get in touch via plosgenetics@plos.org.

# Editor report: Acceptance letter

**DOI:** 10.1371/journal.pgen.1012211.r006

**Authors:** Gregory Cooper, Stuart Scott, Gregory Cooper, Stuart Scott, Gregory Cooper, Stuart Scott

PGENETICS-D-25-01087R2

A genomic-led strategy to anticipate drug safety effects

Dear Dr Pereira,

We are pleased to inform you that your manuscript entitled "

A genomic-led strategy to anticipate drug safety effects" has been formally accepted for publication in PLOS Genetics! Your manuscript is now with our production department and you will be notified of the publication date in due course.

The corresponding author will soon be receiving a typeset proof for review, to ensure errors have not been introduced during production. Please review the PDF proof of your manuscript carefully, as this is the last chance to correct any errors. Please note that major changes, or those which affect the scientific understanding of the work, will likely cause delays to the publication date of your manuscript.

Soon after your final files are uploaded, unless you have opted out or your manuscript is a front-matter piece, the early version of your manuscript will be published online. The date of the early version will be your article's publication date. The final article will be published to the same URL, and all versions of the paper will be accessible to readers.

For Research Articles, you will receive an invoice from PLOS for your publication fee after your manuscript has reached the completed accept phase. If you receive an email requesting payment before acceptance or for any other service, this may be a phishing scheme. Learn how to identify phishing emails and protect your accounts at https://explore.plos.org/phishing.

Thank you again for supporting PLOS Genetics and open-access publishing. We are looking forward to publishing your work!

With kind regards,

Anitha Samidurai

PLOS Genetics

On behalf of:

The PLOS Genetics Team

Carlyle House, Carlyle Road, Cambridge CB4 3DN | United Kingdom

plosgenetics@plos.org | +44 (0) 1223-442823

plosgenetics.org | Twitter: @PLOSGenetics
